# Acidic Activated Charcoal Prevents Obesity and Insulin Resistance in High-Fat Diet-Fed Mice

**DOI:** 10.3389/fnut.2022.852767

**Published:** 2022-05-12

**Authors:** Xuguang Zhang, Pan Diao, Hiroaki Yokoyama, Yoshiki Inoue, Kazuhiro Tanabe, Xiaojing Wang, Chihiro Hayashi, Tomoki Yokoyama, Zhe Zhang, Xiao Hu, Takero Nakajima, Takefumi Kimura, Jun Nakayama, Makoto Nakamuta, Naoki Tanaka

**Affiliations:** ^1^Department of Metabolic Regulation, Shinshu University School of Medicine, Matsumoto, Japan; ^2^Sumi Plus Lab Co., Ltd., Yokohama, Japan; ^3^Ina Carbonization Laboratory Co., Ltd., Ina, Japan; ^4^Medical Solution Promotion Department, Medical Solution Segment, LSI Medience Corporation, Tokyo, Japan; ^5^Department of Gastroenterology, Lishui Hospital, Zhejiang University School of Medicine, Lishui, China; ^6^Department of Pathophysiology, Hebei Medical University, Shijiazhuang, China; ^7^Department of Gastroenterology, Shinshu University School of Medicine, Matsumoto, Japan; ^8^Department of Molecular Pathology, Shinshu University School of Medicine, Matsumoto, Japan; ^9^Department of Gastroenterology, Kyushu Medical Center, Fukuoka, Japan; ^10^Department of Global Medical Research Promotion, Shinshu University Graduate School of Medicine, Matsumoto, Japan; ^11^International Relations Office, Shinshu University School of Medicine, Matsumoto, Japan; ^12^Research Center for Social Systems, Shinshu University, Matsumoto, Japan

**Keywords:** obesity, acidic activated charcoal, insulin resistance, metabolic syndrome, metabolomic analysis, bile acid, intestine, enterohepatic circulation

## Abstract

Obesity is becoming a major public health problem worldwide. Making charcoal from wood (“Sumi-yaki”) has been a traditional activity in the southern part of Nagano Prefecture for centuries, with activated charcoal having reported detoxifying effects. However, it is unclear whether activated charcoal also possesses anti-obesity properties. Additionally, since activated charcoal is usually alkaline and might be affected by gastric juice, we evaluated the effect of acidic activated charcoal on high-fat diet (HFD)-induced obesity. This study demonstrated that co-treatment of acidic activated charcoal with a HFD significantly improved obesity and insulin resistance in mice in a dose-dependent manner. Metabolomic analysis of cecal contents revealed that neutral lipids, cholesterol, and bile acids were excreted at markedly higher levels in feces with charcoal treatment. Moreover, the hepatic expressions of genes encoding cholesterol 7 alpha-hydroxylase and hydroxymethylglutaryl-CoA reductase/synthase 1 were up-regulated by activated charcoal, likely reflecting the enhanced excretions from the intestine and the enterohepatic circulation of cholesterol and bile acids. No damage or abnormalities were detected in the gastrointestinal tract, liver, pancreas, and lung. In conclusion, acidic activated charcoal may be able to attenuate HFD-induced weight gain and insulin resistance without serious adverse effects. These findings indicate a novel function of charcoal to prevent obesity, metabolic syndrome, and related diseases.

## Introduction

Obesity has reached pandemic levels worldwide. Since 1980, the prevalence of obesity has doubled in more than 70 countries/regions and has continued to increase in most other countries. Obesity is a disease caused by excessive fat accumulation. It increases the risk of other diseases, such as cardiovascular disease, type 2 diabetes, hypertension, non-alcoholic fatty liver disease, chronic kidney disease, sleep apnea syndrome, and mood disorders. In recent years, obesity has become a major public health concern in South Asia. In some Asian countries, obesity and its related metabolic diseases is widespread in more than one-third of the population (1–3).

Making charcoal from wood (“Sumi-yaki”) has been a traditional activity in the southern part of Nagano Prefecture for centuries, with “Ina Akamatsu” charcoal was made into activated charcoal for deodorant and water purification. Previous studies have revealed detoxifying effects for activated charcoal. Meinita et al. reported that the material could remove 5-hydroxymethylfurfural, levulinic acid, and other toxic substances within 30 min (4). Elsewhere, recommendations given by poison control centers in Germany state that activated charcoal is suitable for primary toxin clearance in cases of moderate-to-severe poisoning; charcoal is especially suitable for poisons that remain for long periods in the stomach and poisons circulating between the intestine and liver (5). Neuvonen et al. also demonstrated the ability of activated charcoal to effectively bind bile acids (BAs) *in vitro* (6). The above observations corroborate the biological action of activated charcoal as an absorber of toxins and lipid derivatives.

It is well known that dietary composition, including fat and contaminants, as well as microbiota-produced BAs affect adiposity through multiple pathways. To date, however, the anti-obesity effect of activated charcoal has not been addressed. In a preliminary experiment, the anti-obesity effect of alkaline activated charcoal was not significant in high-fat diet (HFD)-treated mice. Additionally, the mice consuming alkaline activated charcoal looked irritable (unpublished data). We speculated that acidic activated charcoal may avoid interference of gastric juice and be more suitable than regular alkaline charcoal. Therefore, we originally developed acidic activated charcoal with a pH of approximately 5 and treated C57BL/6J mice with the acidic activated charcoal powder-containing HFD, in order to investigate whether the acidic charcoal exerts an anti-obesity effect.

## Materials and Methods

### Mice and Treatment

All animal experiments were conducted in adherence to the animal research methods outlined in the “Guidelines for the Care and Use of Laboratory Animals” approved by the ethics committee of Shinshu University School of Medicine. Male 6-week-old mice on a C57BL/6J genetic background were purchased from CLEA Japan, Inc. (Tokyo, Japan) and maintained in a controlled and clean environment (25°C, 12-h light/dark cycle). The control diet was purchased from ORIENTAL YEAST Co., Ltd (Tokyo, Japan). The HFD (D12492M) containing 60% kcal of fat was purchased from Research Diet (New Brunswick, NJ). The ingredients of these diets were shown in Supplementary Tables 1, 2, respectively. Acidic activated charcoal was provided from Sumi Plus Lab Co., Ltd. (Yokohama, Kanagawa, Japan), with dextrin (Pinedex#2, Matsutani Chemical Industry Co., Ltd., Itami, Hyogo, Japan) used as a vehicle. The acidic charcoal was prepared by carbonizing plants and trees at constant atmospheric pressure and temperature above 700°C after soaking acidic deep seawater (pH 5.5). The activated charcoal powder was completely mixed with HFD and then administered to mice. Inulin (Fuji FF, Fuji Nihon Seito Corporation, Tokyo, Japan) and raffinose (Nitten Raffinose FP, Nippon Beet Sugar Manufacturing Co., Ltd., Tokyo, Japan) were added to prevent charcoal-induced constipation. In the first experiment, mice (*n* = 21) weighing 22–26 grams were randomly divided into three groups after acclimatization for at least 2 weeks into (1) a control diet group (Con, *n* = 5), (2) a HFD with 5% dextrin and 5% inulin raffinose group (HFD + Veh, *n* = 8), and (3) a HFD with 5% acidic activated charcoal and 5% inulin raffinose group (HFD + 5%C, *n* = 8). As an additional dose-dependency experiment, mice (*n* = 28) weighing 23–28 grams were randomly divided into five groups fed (1) a control diet (Con, *n* = 4), (2) a HFD with 5% dextrin and 5% inulin raffinose (HFD + Veh, *n* = 6), (3) a HFD with 1.5% acidic activated charcoal and 1.5% inulin raffinose (HFD + 1.5%C, *n* = 6), (4) a HFD with 3% acidic activated charcoal and 3% inulin raffinose (HFD + 3%C, *n* = 6), and (5) a HFD with 4.5% acidic activated charcoal and 4.5% inulin raffinose (HFD + 4.5%C, *n* = 6). During the experiments, the animals’ body weight (BW) and food intake were measured weekly. At 12 weeks of treatment, the mice were sacrificed by CO_2_ asphyxiation after 6-h fasting for collecting blood and tissues. Blood samples were centrifuged twice at 3,000 rpm for 15 min to obtain serum and stored at –80°C until use. The organs were harvested, washed with 0.9% sterile saline, and weighed, and the length of the small intestine was measured. The tissues were preserved either by immediate fixation in 10% neutral formalin or immediate freezing with dry ice and storage at –80°C. The feces during 6-h fasting before killing were collected manually, weighted, and stored at –80°C until use.

### Glucose/Insulin Tolerance Tests

For the glucose tolerance test (GTT), the mice were injected intraperitoneally with 1 g/kg BW of glucose dissolved in 0.9% sterile saline using a 26-gauge needle after 18-h fasting. For the insulin tolerance test (ITT), the mice were injected intraperitoneally with 0.6 unit/kg BW of insulin (Humulin R; Eli Lilly, Indianapolis, IN) in the same manner after 6-h fasting. Blood glucose concentrations were determined at 0, 15, 30, 60, 90, and 120 min after injection using a FreeStyle Freedom Lite glucose meter (NIPRO, Osaka, Japan).

### Biochemical Analysis

Serum levels of aspartate aminotransferase (AST) (#431-30901), alanine aminotransferase (ALT) (#431-30901), triglycerides (TGs) (#632-50991), total cholesterol (T-Chol) (#294-65801), non-esterified fatty acids (NEFAs) (#279-75401), phospholipids (PLs) (#433-36201), total bile acid (TBA) (#431-15001), and glucose (#298-65701) were measured with commercially available enzyme assay kits (Wako Pure Chemical Industries Co., Ltd., Osaka, Japan). Serum insulin, leptin, and high-molecular-weight adiponectin were determined by ELISA kits (AKRIN-011T, AKRLP-011, and AKMAN-011, respectively, FUJIFILM Wako Shibayagi, Gunma, Japan). Homeostatic model assessment for insulin resistance (HOMA-IR) was calculated using the equations published by Matthews et al. (7). Serum thyroid-stimulating hormone (TSH) was determined by ELISA kits (KT-29922, KAMIYA BIOMEDICAL COMPANY, Seattle, WA) (8).

Total liver lipids and fecal lipids were extracted according to the hexane:isopropanol method (9) with a slight modification and quantified using the abovementioned kit (Wako Pure Chemical Industries Co., Ltd.). For extracting liver lipids, approximately 50 mg of liver tissues were disrupted and sonicated in 5–10 volumes of sodium phosphate buffer (NaPi, 50 mM). The lysate (50 μL) was drawn into a new tube, and 900 μL of hexane/isopropanol (3:2, vol/vol) (HIP) was added. The tube was vigorously vortexed for 1 min and centrifuged at 2,500 rpm for 5 min at 4°C, and the upper layer was transferred to a new tube and centrifuged under vacuum at 40–50°C. After evaporation, the lipid extracts were dissolved in 100 μL of HIP containing 1% (wt/vol) Triton X-100 and evaporated again by vacuum centrifuge. Then, 100 μL of distilled water was added to the sample, incubated at 37°C for 30 min, and vortexed. Finally, the lipid extracts were solubilized in 1% Triton X-100/water as described previously (10), and 10 μL of the lipid extracts (corresponding to 0.25 mg of livers) were used for measuring TG, PL, T-Chol, and NEFAs (11).

For extracting fecal lipids, 20–30 mg of dry feces were collected and placed in 19 volumes of 50 mM NaPi. The tube was kept on ice for 30–60 min until the feces become swollen, and then sonicated on ice until the feces were completely dispersed in the solution. The total lipids in feces were extracted in a similar manner as those in the liver and finally solubilized in 1% Triton X-100/water at a concentration of 0.025 mg feces eq./μL. Ten μL of the lipid extracts (corresponding to 0.25 mg of feces) were used for measuring TBA, TG, T-Chol, PL, and NEFAs.

### Histological Analysis

Formalin-fixed liver tissues were embedded in paraffin, cut into 3 μm sections, and stained with hematoxylin and eosin (12). To quantify the size of adipocytes and the relative area of islets, 10 independent sections of epididymal white adipose tissue (eWAT) and pancreas were photomicrographed (×200 magnification) in each group, and the size of each adipocyte and the area of each islet were quantified using the 1.52V version of Image J (National Institutes of Health, Bethesda, MD). Values were expressed as the percentage of adipocytes of the corresponding size. The total islet area was also expressed as the percentage of the total area of pancreatic parenchyma in the sections.

### Quantification of mRNA Levels

Total RNA was isolated from frozen liver tissues using a NucleoSpin RNA Plus Kit (QiagenMACHEREY-NAGEL Gmbh & Co., KG, Neumann, Germany). Total RNA from the stomach, upper small intestine, lower small intestine, large intestine, brown adipose tissue (BAT), and eWAT were extracted using TRI reagent (MOR Molecular Research Center, Inc., Cincinnati, OH). Tissues (50 mg) were disrupted and sonicated in 500 μL of TRI reagent. The homogenate was incubated at room temperature for 5 min, 100 μL of chloroform was added, and the homogenate was shaken vigorously for 15 sec. The homogenate was then incubated at room temperature for 2–3 min and centrifuged at 12,000 × *g* for 15 min at 4°C. The upper layer was transferred to a new tube and 1 volume of 70% ethanol was added and vortexed. The mixture was transferred to a column with a silica membrane and centrifuged at 12,000 × *g* for 1 min at room temperature. The silica membrane was washed and dried, and RNA was eluted and collected using RNase-free water. The RNA samples were reverse transcribed to cDNA using ReverTra Ace qPCR RT Master Mix (Toyobo Co., Ltd., Osaka, Japan). All mRNA levels were determined by the real-time quantitative polymerase chain reaction (qPCR) using a SYBR qPCR mix (Toyobo Co., Ltd.) on a Thermo Fisher QuantStudio 3 Real-Time PCR Instrument (Thermo Fisher Scientific, Waltham, MA). The mRNA levels were normalized to 18S ribosomal RNA (18S rRNA) levels and expressed as fold changes relative to those of C57BL/6J mice fed the control diet. The primer sequences were listed in Supplementary Table 3.

### Metabolomic Analysis

The detailed method of metabolomic analysis is described in Supplementary Information.

### Statistical Analysis

Results were expressed as the mean ± standard error of the mean (SEM). Two-tailed Student’s *t*-tests and a one-way analysis of variance (ANOVA) with the Bonferroni’s correction were conducted using SPSS statistics version 22 (IBM, Armonk, NY). A *P*-value of less than 0.05 was considered statistically significant.

## Results

### Acidic Activated Charcoal Prevents High-Fat Diet-Induced Obesity

**FIGURE 1:**
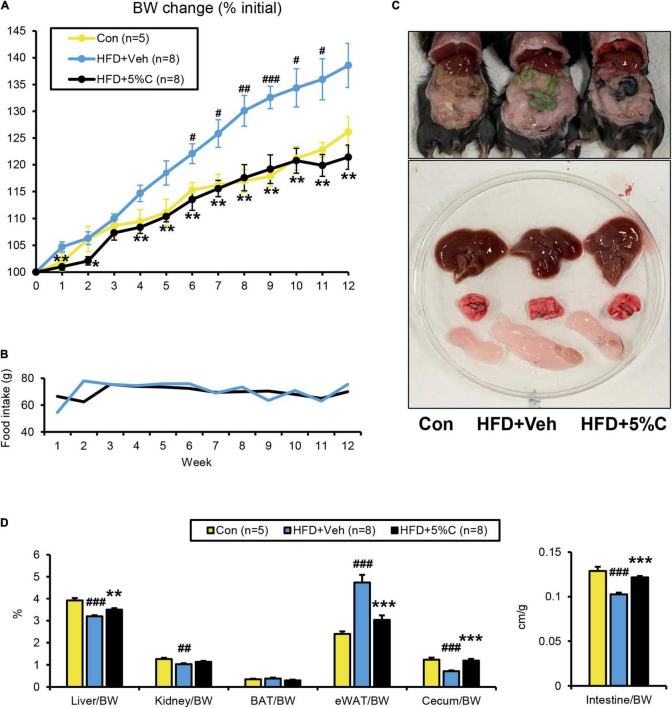
A diet containing acidic activated charcoal can improve obesity in mice fed HFD after 12 weeks of treatment. **(A)** Changes in BW. **(B)** Food intake. **(C)** Gross appearance of representative mice from the three groups. From top to bottom, the liver, lung and epididymal white adipose tissue (eWAT) are shown. **(D)** The weights of the liver, kidney, BAT, eWAT, and cecum along with the length of the small intestine. Values were corrected by body weight (BW). Data are expressed as the mean ± SEM. Statistical analysis was performed using two-tailed Student’s *t*-tests. *P < 0.05, **P < 0.01, and ***P < 0.001 between the HFD + Veh group and the HFD + 5%C group. ^#^*P* < 0.05, ^##^*P* < 0.01, and ^###^*P* < 0.001 between the HFD + Veh group and the Con group.

In the first experiment, test mice were given the three different diets for 12 weeks. In the HFD + 5%C group, the limbs, fur and tail turned black from the charcoal (Supplementary Figure 1A). Weight gain in the HFD + 5%C group was significantly suppressed as compared with the HFD + Veh group and was similar to the Con group (Figure 1A and Supplementary Table 4). There were no significant differences in food intake between the HFD + Veh and HFD + 5%C groups (Figure 1B), nor were remarkable differences seen for the gross appearance of the liver, lungs, or subscapular BAT among the three groups (Figures 1C,D). We observed that eWAT became larger, the cecum became smaller, and the small intestine became shorter by HFD treatment, which were all mitigated by the charcoal-containing HFD (Figures 1C,D and Supplementary Table 4). Since thyroid dysfunction can lead to darker fur and weight loss, we measured serum levels of TSH, a sensitive indicator of primary hypothyroidism. The TSH levels were not changed by charcoal treatment (Supplementary Figure 1B). These results demonstrated that acidic activated charcoal prevented HFD-induced obesity.

### Acidic Activated Charcoal Improves High-Fat Diet-Induced Insulin Resistance

**FIGURE 2:**
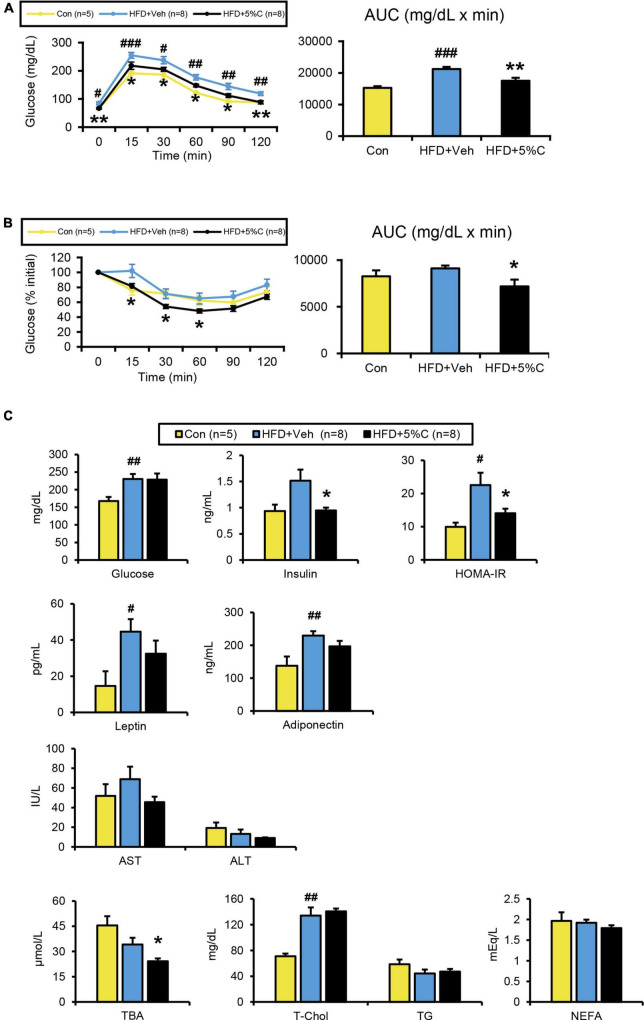
Changes in metabolic parameters of mice treated with acidic activated charcoal. **(A)** GTT at 10 weeks of HFD feeding. **(B)** ITT at 12 weeks of HFD feeding. **(C)** Serum levels of glucose, insulin, HOMA-IR, leptin, high-molecular-weight adiponectin, AST, ALT, TBA, T-Chol, TG, and NEFA. Data are expressed as the mean ± SEM. Statistical analysis was performed using two-tailed Student’s *t*-tests. *P < 0.05 and **P < 0.01 between the HFD + Veh group and the HFD + 5%C group. ^#^*P* < 0.05, ^##^*P* < 0.01, and ^###^*P* < 0.001 between the HFD + Veh group and the Con group.

Since obesity has been found to impair glucose metabolism, the GTT was conducted in the tenth week of HFD treatment. The blood glucose levels of the HFD + 5%C group were consistently lower than those of the HFD + Veh group (Figure 2A). Accordingly, the area under the receiver operating characteristic curve (AUC) was significantly smaller in the HFD + 5%C group than in the HFD + Veh group (Figure 2A).

To assess whether the improved glucose intolerance stemmed from enhanced insulin sensitivity, the ITT was performed in the twelfth week of HFD treatment. After insulin injection, glucose levels in the HFD + 5%C group fell faster than in the HFD + Veh group (Figure 2B). The blood glucose initial ratio in the HFD + 5%C group was consistently lower than in the HFD + Veh group. Consequently, the AUC was also significantly smaller in the HFD + 5%C group (Figure 2B). These results indicated that acidic activated charcoal intake could reverse HFD-induced insulin resistance.

In agreement with the GTT/ITT results, serum insulin levels and HOMA-IR were significantly lower in the HFD + 5%C group than in the HFD + Veh group, while serum leptin and high-molecular-weight adiponectin were comparable between the HFD + Veh and HFD + 5%C groups (Figure 2C). To exclude the possibility that decreased serum insulin levels were due to impaired pancreatic function, we assessed the histology of pancreas. There were no significant abnormalities, such as atrophy, inflammation, and fibrosis, in pancreatic parenchyma of charcoal-containing HFD-fed mice. Quantification of the relative islet area and pancreatic expression levels of *Ins2* (insulin 2) revealed no significant differences between the groups (Supplementary Figures 2A,B).

In the determination of serum lipid profiles, while serum TBA levels were significantly decreased in the HFD + 5%C group, no significant differences in the other parameters were observed between the HFD + Veh and HFD + 5%C groups (Figure 2C).

### Acidic Activated Charcoal Has No Obvious Effect on the Liver

In both the HFD + Veh and HFD + 5%C groups, the serum levels of AST and ALT, which are conventional indices of liver damage, were within the reference values, and no abnormal morphologies were found in the liver tissue (Figure 2C and Supplementary Figure 3A). In the latter group, no charcoal deposition was observed in the liver. Quantification of mRNA in the liver demonstrated no significant differences in gene expression related to fatty acid (FA)/TG metabolism, inflammation, or fibrosis (Supplementary Figures 3B–F). Quantitative analysis of hepatic lipids showed that TGs in the HFD + 5%C group were increased, while PL, T-Chol, and NEFA levels were comparable among the groups (Supplementary Figure 3G). Taken together, it appeared that the liver was not a primary target of acidic activated charcoal for preventing HFD-induced BW gain and insulin resistance.

### Acidic Activated Charcoal Improves Epididymal White Adipose Tissue Hypertrophy and Inflammation Caused by High-Fat Diet

**FIGURE 3:**
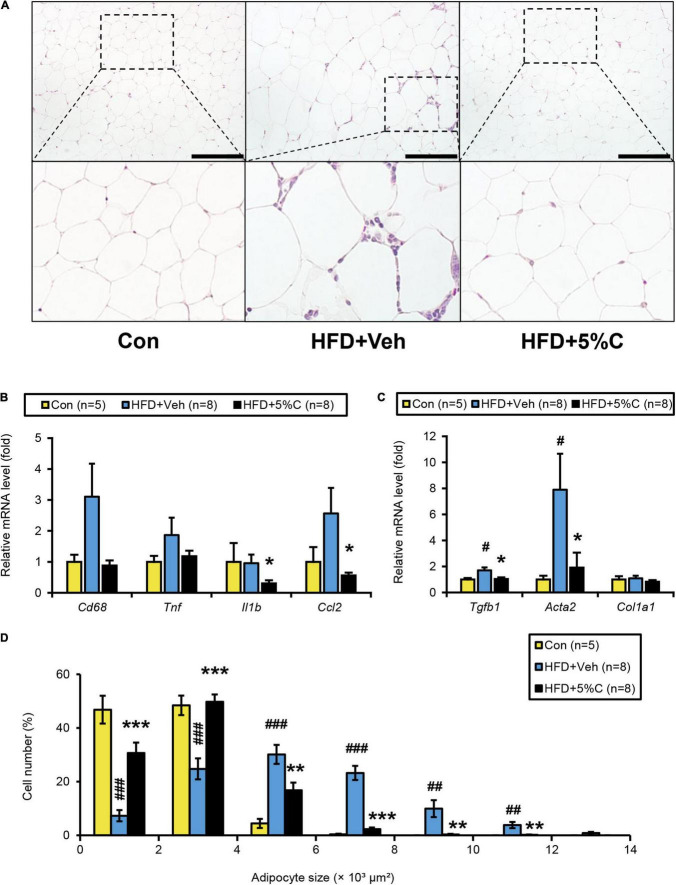
Changes in eWAT after activated charcoal treatment. **(A)** Histological appearance of eWAT. The sections were stained with hematoxylin and eosin. Scale bars = 150 μm (x200 magnification). **(B)** The mRNA levels of genes related to inflammation (*Cd68, Tnf, Il1b*, and *Ccl2*) in the liver. **(C)** The mRNA levels of genes related to fibrosis (*Tgfb1, Acta2*, and *Col1a1*) in the liver. **(D)** Proportions of adipocytes of different sizes. Data are expressed as the mean ± SEM. Statistical analysis was performed using two-tailed Student’s *t*-tests. *P < 0.05, **P < 0.01, and ***P < 0.001 between the HFD + Veh group and the HFD + 5%C group. ^#^*P* < 0.05, ^##^*P* < 0.01, and ^###^*P* < 0.001 between the HFD + Veh group and the Con group.

The finding that acidic activated charcoal prevented eWAT weight gain prompted us to assess the phenotypic changes in white adipocytes. Microscopic examination of eWAT revealed marked adipocyte hypertrophy and inflammatory cell infiltration around adipocytes in the HFD + Veh group, and the proportion of larger adipocytes (4,001–12,000 μm^2^) in this group was significantly increased. These alterations were prevented by 5% charcoal intake (Figures 3A,D).

We next evaluated whether eWAT mRNA levels were associated with adipocyte enlargement and inflammation. Compared with the HFD + Veh group, the expression levels of the genes related to inflammation and fibrosis, including interleukin 1 beta (*Il1b*), chemokine (C-C motif) ligand 2 (*Ccl2*), transforming growth factor beta 1 (*Tgfb1*), and actin alpha 2 (*Acta2*), were significantly decreased in the HFD + 5%C group (Figures 3B,C). The mRNA levels of other inflammation-related genes, such as cluster of differentiation 68 (*Cd68*) and tumor necrosis factor alpha (*Tnf*), were decreased as well but did not reach statistical significance (Figures 3B,C).

The improvement of adipocyte hypertrophy in the HFD + 5%C group may have been associated with increased FA catabolism, browning of white adipocytes, and decreased FA uptake and TG synthesis. Among several genes related to FA/TG metabolism, the mRNA levels of the cluster of differentiation 36 (*Cd3*6) and FA-binding protein 4 (*Fabp4*) genes, both responsible for FA uptake into adipocytes, tended to be decreased in the HFD + 5%C group (Supplementary Figure 4). No browning of white adipocytes was detected in the HFD + 5%C group (Supplementary Figure 4).

### Acidic Activated Charcoal Improves Whitening of Brown Adipose Tissue Caused by High-Fat Diet, but Does Not Enhance Browning

BAT plays an important role in the maintenance of whole-body energy/fuel metabolism through the promotion of fat burning. To evaluate whether the anti-obesity effect of charcoal stemmed from enhanced fat burning in BAT, we assessed for phenotypic changes in this tissue. Histological analysis revealed that the HFD + Veh group had increased unilocular lipid droplets and white adipocytes (i.e., whitening), with slight inflammatory cell infiltration around adipocytes, while such changes were absent in the HFD + 5%C group (Supplementary Figure 5A). In mRNA quantifications of BAT, compared with the HFD + Veh group, the expression levels of the inflammation-related genes *Cd68* and *Ccl2* tended to be decreased in the HFD + 5%C group. The mRNA levels associated with fat burning, including uncoupling protein 1 (*Ucp1*), cell death-inducing DNA fragmentation factor alpha-like effector A (*Cidea*), type 2 iodothyronine deiodinase (*Dio2*), peroxisome proliferator-activated receptor gamma coactivator 1 alpha (*Ppargc1a*), and cytochrome c oxidase subunit 5B (*Cox5b*) and 8B (*Cox8b*), were not increased by the co-administration of charcoal (Supplementary Figure 5B). Cell death-inducing DNA fragmentation factor alpha-like effector C (*Cidec*) as a gene related to lipid storage was decreased but did not reach statistical significance. The above results indicated that although activated charcoal could improve whitening in BAT, it was not due to enhanced fat oxidation.

### Acidic Activated Charcoal Does Not Damage the Lungs and Digestive Tract

Since acidic activated charcoal powder was not digested or absorbed into the body and charcoal was excreted into the feces and mouse bedding in the cage turned black by the charcoal treatment (Supplementary Figure 6A), we next examined for possible pulmonary and gastrointestinal toxicity. To assess the presence of inflammation in the lungs and gastrointestinal tract, we determined mRNA levels in the lung (Supplementary Figure 6B), stomach (Supplementary Figure 6C), upper small intestine (Supplementary Figure 6D), lower small intestine (Supplementary Figure 6E), and large intestine (Supplementary Figure 6F). The mRNA levels of genes related to inflammation [*Il1b*, *Ccl2*, and nitric oxide synthase 2 (*Nos2*)], fibrosis (*Tgfb1*), and neutrophil infiltration [myeloperoxidase (*Mpo*)] were not significantly different between the HFD + 5%C and HFD + Veh groups. The possibility that co-treatment with acidic activated charcoal could injure/irritate the respiratory and gastrointestinal mucosa was therefore considered low.

### Acidic Activated Charcoal Affects Bile Acid Metabolism

**FIGURE 4:**
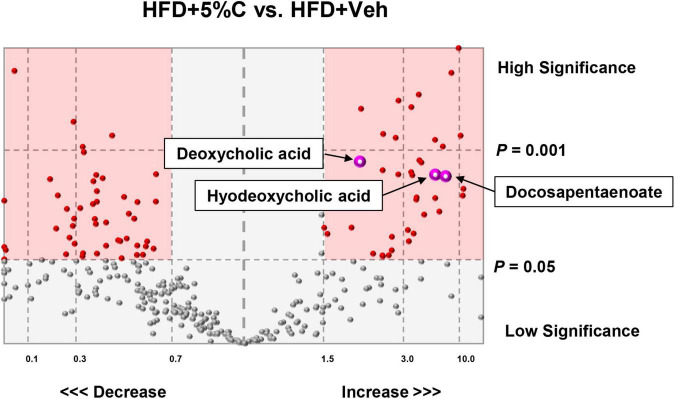
Metabolomic analysis of cecal contents. A volcano plot identified 42 metabolites, including deoxycholic acid, hyodeoxycholic acid, and docosapentaenoate, that were significantly increased in the HFD + 5%C group as compared with the HFD + Veh group (right pink square).

**FIGURE 5:**
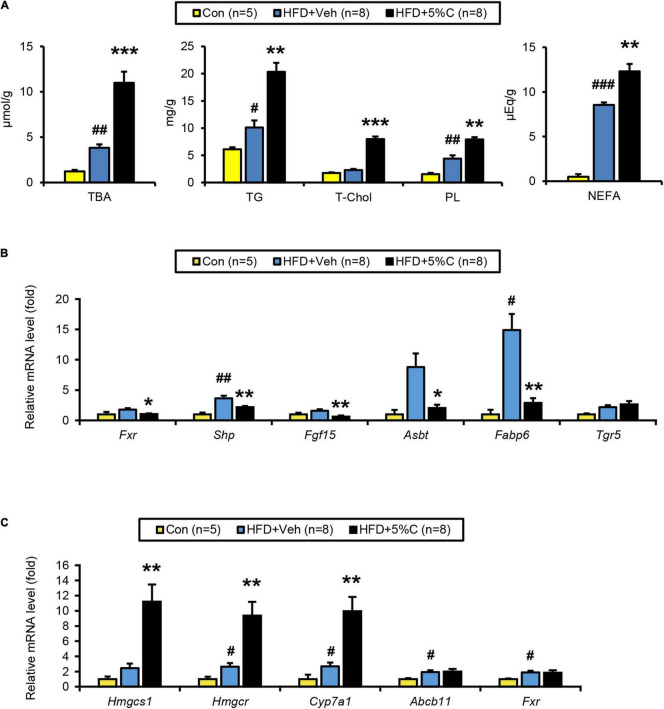
BA metabolism is affected by activated charcoal treatment. **(A)** Quantitative analysis of TBA, TG, T-Chol, PL, and NEFA in cecal contents. **(B)** The mRNA levels of genes related to BA metabolism (*Fxr, Shp, Fgf15, Asbt*, *Fabp6*, and *Tgr5*) in the intestine. **(C)** The mRNA levels of genes related to BA/cholesterol metabolism (*Hmgcs1, Hmgcr*, *Cyp7a1, Abcb11*, and *Fxr*) in the liver. Data are expressed as the mean ± SEM. Statistical analysis was performed using two-tailed Student’s *t*-tests. *P < 0.05, **P < 0.01, and ***P < 0.001 between the HFD + Veh group and the HFD + 5%C group. ^#^*P* < 0.05, ^##^*P* < 0.01, and ^###^*P* < 0.001 between the HFD + Veh group and the Con group.

Since shortening of the small intestine and a reduction in cecal contents were prevented by treatment with acidic activated charcoal, we tested the possibility that the intestinal environment was altered by such treatment by the metabolomic analysis of cecal contents. A volcano plot identified 42 metabolites that were significantly increased in the HFD + 5%C group as compared with the HFD + Veh group (Figure 4). The 20 most significantly increased metabolites are listed in Supplementary Table 5. Nine metabolites were neutral lipids, FAs, or BAs such as docosapentaenoate, deoxycholic acid (DCA), and hyodeoxycholic acid (Figure 4). To validate these metabolomic results, fecal lipids and BAs were quantified, revealing that the amounts of TGs, PLs, and TBA were significantly increased in the feces of all mice in the HFD + 5%C group (Figure 5A).

BA metabolism, including DCA, lithocholic acid, and taurocholic acid, is controlled by farnesoid X receptor (FXR). Analysis of the FXR signaling pathway in the lower small intestine and liver revealed that the mRNA levels of *Fxr* and its target genes, including small heterodimer partner (*Shp*), fibroblast growth factor 15 (*Fgf15*), apical sodium BA transporter (*Asbt*), and FA-binding protein 6 (*Fabp6*), were significantly down-regulated in the HFD + 5%C group vs. the HFD + Veh group in the intestine (Figure 5B) while those of *Fxr*-regulated genes were unchanged in the liver, indicating that intestinal FXR was specifically down-regulated by charcoal co-administration. BAs have also been shown to effectively activate Takeda G protein-coupled receptor 5 (TGR5), although the mRNA levels of *Tgr5* in the lower small intestine were not significantly increased in the HFD + 5%C group (Figure 5B).

The hepatic mRNA expression of cholesterol 7 alpha-hydroxylase (*Cyp7a1*), a rate-limiting enzyme of BA synthesis, as well as those of hydroxymethylglutaryl-CoA synthase 1 (*Hmgcs1*) and reductase (*Hmgcr*), both rate-limiting enzymes of *de novo* cholesterol synthesis, were significantly increased in the HFD + 5%C group as compared with the HFD + Veh group, while those of ATP-binding cassette sub-family B member 11 (*Abcb11*) and *Fxr* were comparable to the HFD + Veh group (Figure 5C). These changes were likely due to an adaptive response to decreased BA reabsorption in the intestine, i.e., increased BA excretion into the feces, by acidic activated charcoal.

### Acidic Activated Charcoal Prevents High-Fat Diet-Induced Obesity and Insulin Resistance in a Dose-Dependent Manner

**FIGURE 6:**
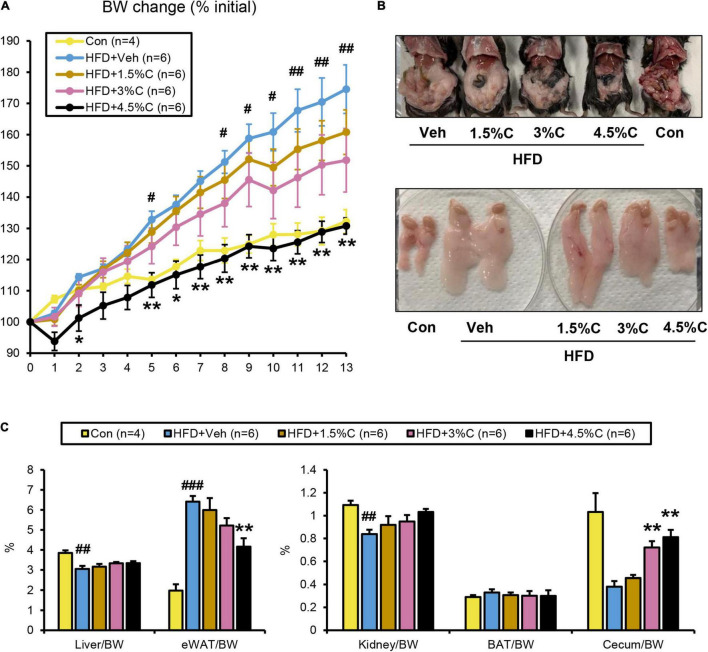
HFD containing 4.5% acidic activated charcoal showed the strongest anti-obesity effect after 13 weeks of treatment. **(A)** Changes in BW. **(B)** Gross appearance of abdominal organs (upper panel) and epididymal white adipose tissue (eWAT) (lower panel) derived from five representative groups of mice. **(C)** Weights of the liver, eWAT, kidney, BAT, and cecum. Values were corrected by body weight (BW). Data are expressed as the mean ± SEM. Statistical analysis was performed using a one-way analysis of variance (ANOVA) with the Bonferroni’s correction. *P < 0.05, and **P < 0.01 between the HFD + Veh group and the HFD + C group. ^#^*P* < 0.05, ^##^*P* < 0.01, and ^###^*P* < 0.001 between the HFD + Veh group and the Con group.

To evaluate the reproducibility and dose dependency of the anti-obesity effect of acidic activated charcoal, a second mouse cohort was given a HFD supplemented with different charcoal contents for 12 weeks. In the 1.5, 3, and 4.5% charcoal-containing HFD groups (HFD + 1.5%C, HFD + 3%C, and HFD + 4.5%C, respectively), weight gain was significantly suppressed vs. the HFD + Veh group, which was enhanced with charcoal dosage (Figure 6A and Supplementary Table 6). There were no significant differences in the gross appearance of the liver or the liver/body weight ratio among the five groups (Figures 6B,C). As in the first experiment, eWAT became larger and the cecum became smaller in the HFD + Veh group over the Con group. Acidic activated charcoal improved these changes in a dose-dependent manner without affecting thyroid function (Figures 6B,C, Supplementary Figure 1B and Supplementary Table 6).

**FIGURE 7:**
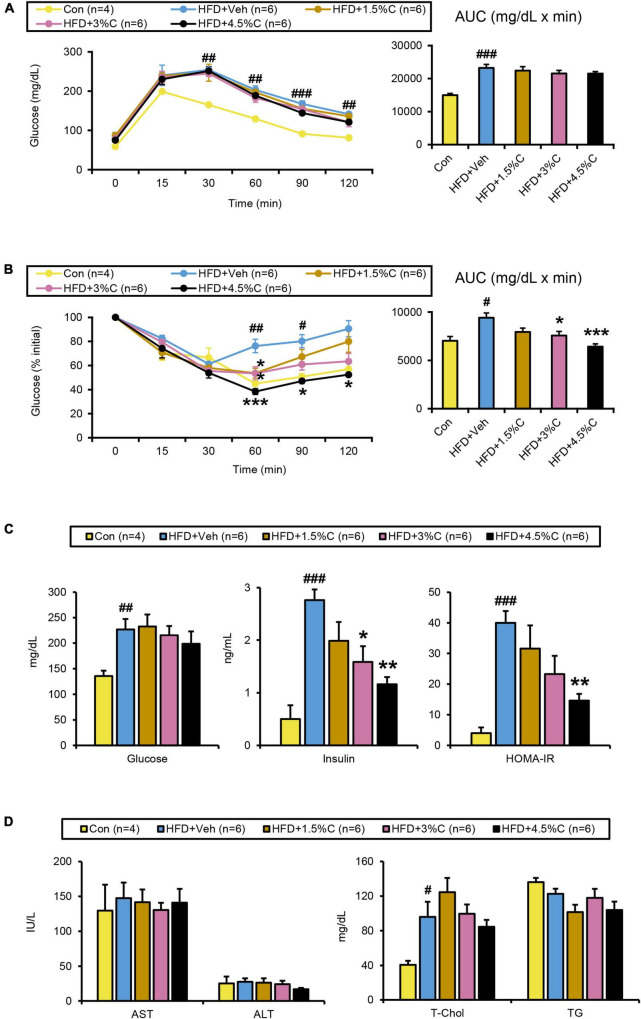
Changes in metabolic parameters of mice treated with different dosages of acidic activated charcoal. **(A)** GTT at 9 weeks of HFD feeding. **(B)** ITT at 11 weeks of HFD feeding. **(C)** Serum levels of glucose, insulin and HOMA-IR. **(D)** Serum levels of AST, ALT, T-Chol, and TG. Data are expressed as the mean ± SEM. Statistical analysis was performed using a one-way analysis of variance (ANOVA) with the Bonferroni’s correction. *P < 0.05, **P < 0.01, and ***P < 0.001 between the HFD + Veh group and the HFD + C group. ^#^*P* < 0.05, ^##^*P* < 0.01, and ^###^*P* < 0.001 between the HFD + Veh group and the Con group.

The GTT was conducted in the ninth week of HFD treatment. The blood glucose levels of all HFD + C (1.5%C, 3%C, and 4.5%C) groups were consistently lower than that of the HFD + Veh group, with the HFD + 4.5%C group exhibiting the lowest blood sugar level (Figure 7A). The AUC became smaller with increasing doses in the HFD + C groups vs. the HFD + Veh group (Figure 7A).

The ITT in the eleventh week of treatment showed that the glucose levels in all HFD + C groups fell faster than in the HFD + Veh group. The decrease in the HFD + 4.5%C group was especially larger than in the Con group (Figure 7B), which was reflected in the AUC results (Figure 7B). Serum insulin levels and HOMA-IR significantly decreased in the HFD + C groups with rising charcoal doses (Figure 7C). These findings indicated that 4.5%C exerted the strongest therapeutic effect on HFD-induced insulin resistance. Similarly to the results of the first experiment, there were no significant differences in serum and hepatic lipid profile parameters between the HFD + Veh and HFD + C groups (Figure 7D and Supplementary Figure 7A).

### Acidic Activated Charcoal Improves High-Fat Diet-Induced Epididymal White Adipose Tissue Hypertrophy and Inflammation as Well as Brown Adipose Tissue Whitening in a Dose-Dependent Manner

**FIGURE 8:**
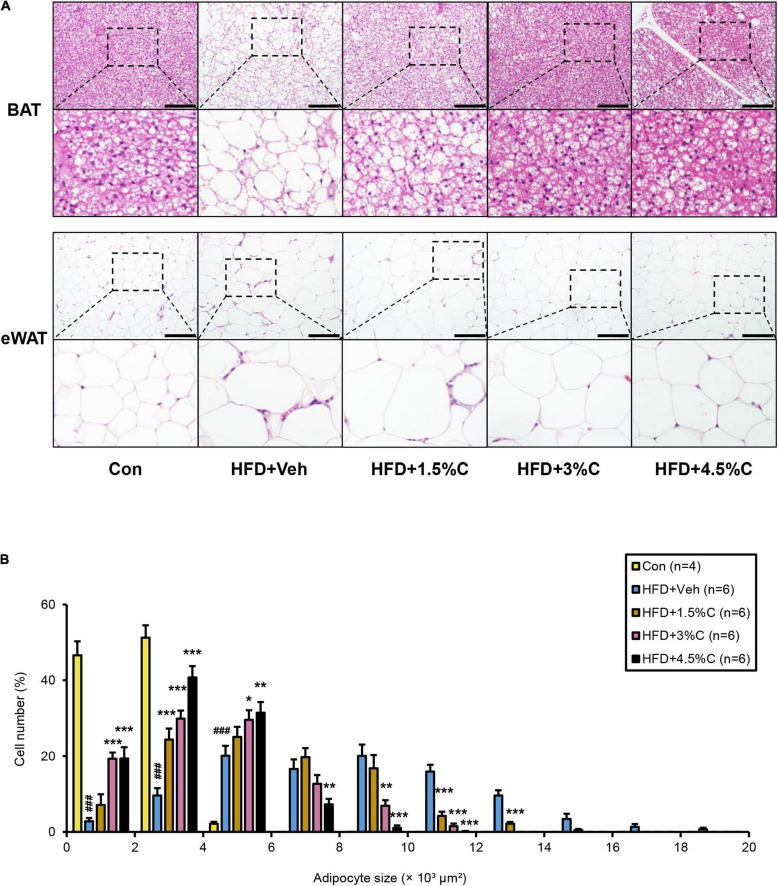
Changes in histological appearance after treatment with different dosages of acidic activated charcoal. **(A)** Histological appearance of BAT and eWAT. The sections were stained with hematoxylin and eosin. Scale bars = 150 μm (x200 magnification). **(B)** Proportions of adipocytes of different sizes. Data are expressed as the mean ± SEM. Statistical analysis was performed using a one-way analysis of variance (ANOVA) with the Bonferroni’s correction. *P < 0.05, **P < 0.01, and ***P < 0.001 between the HFD + Veh group and the HFD + C group. ^###^*P* < 0.001 between the HFD + Veh group and the Con group.

**FIGURE 9:**
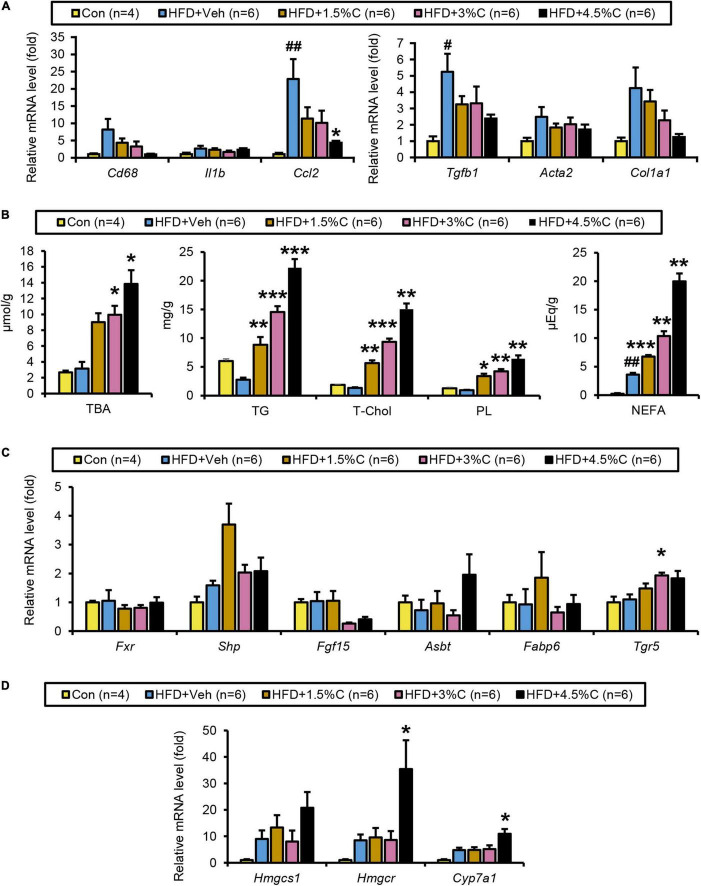
Changes in lipid metabolism after treatment with different dosages of acidic activated charcoal. **(A)** The mRNA levels of genes related to inflammation (*Cd68, Il1b*, and *Ccl2*) and fibrosis (*Tgfb1, Acta2*, and *Col1a1*) in the liver. **(B)** Quantitative analysis of TBA, TG, T-Chol, PL, and NEFA in cecal contents. **(C)** The mRNA levels of genes related to BA metabolism (*Fxr, Shp, Fgf15, Asbt, Fabp6*, and *Tgr5*) in the intestine. **(D)** The mRNA levels of genes related to cholesterol metabolism (*Hmgcs1, Hmgcr*, and *Cyp7a1*) in the liver. Data are expressed as the mean ± SEM. Statistical analysis was performed using a one-way analysis of variance (ANOVA) with the Bonferroni’s correction. *P < 0.05, **P < 0.01, and ***P < 0.001 between the HFD + Veh group and the HFD + C group. ^#^*P* < 0.05, and ^##^*P* < 0.01 between the HFD + Veh group and the Con group.

Microscopic examination of eWAT revealed that the adipocyte hypertrophy and inflammatory cell infiltration around adipocytes in the HFD + Veh group were dose-dependently corrected by charcoal intake; as the charcoal doses increased, the proportion of larger adipocytes decreased significantly (Figures 8A,B). Compared with the HFD + Veh group, the expression levels of inflammation- and fibrosis-related genes, such as *Cd68*, *Il1b*, *Ccl2*, *Tgfb1*, *Acta2*, and *Col1a1*, were decreased in the HFD + C groups with rising charcoal doses (Figure 9A). Histological analysis of BAT revealed that the HFD-induced unilateral lipid droplets and slight inflammatory cell infiltration around adipocytes were ameliorated with increasing charcoal doses (Figure 8A). Overall, our results indicated that the improvement of HFD-induced eWAT hypertrophy and inflammation by acidic activated charcoal, as well as BAT whitening, were dose-dependent.

### Acidic Activated Charcoal Increases Fecal Lipids and Bile Acids in a Dose-Dependent Manner

The amounts of TBA, TGs, T-Chol, PLs, and NEFAs all significantly increased with higher charcoal doses in feces samples collected from the HFD + C groups (Figure 9B). However, *Fxr* and its related genes (*Shp*, *Fgf15*, *Asbt*, and *Fabp6*) showed no remarkable differences between the HFD + Veh group and the HFD + C groups. Interestingly, the mRNA level of *Tgr5* increased with greater doses of charcoal, which was significant in the HFD + 3%C groups (Figure 9C).

The finding that the hepatic mRNA expressions of *Hmgcr* and *Cyp7a1* were significantly increased in the HFD + 4.5%C group indicated enhanced synthesis of cholesterol and BAs in the liver (Figure 9D), likely reflecting dose-dependent increases in cholesterol/BA excretion into the feces.

## Discussion

The current study showed that acidic activated charcoal could prevent HFD-induced obesity, insulin resistance, eWAT hypertrophy and inflammation, as well as enhance BAT whitening in a dose-dependent manner without any serious adverse effects. Metabolomic analysis of cecal contents revealed that neutral lipids, cholesterol, and BAs were markedly more excreted into the feces with charcoal supplementation. Consequently, the enterohepatic circulation of cholesterol/BA was promoted as evidenced by the up-regulated hepatic expression of *de novo* BA- and cholesterol-synthesizing enzymes (13). The current study proposes a novel function of acidic activated charcoal to prevent obesity, overnutrition, metabolic syndrome, and related diseases.

The most intriguing finding of the initial study was that the BW gain in the mice treated with activated charcoal was significantly lower than in the HFD + Veh group, which was similar to that in the Con group, with no difference in food intake or thyroid function between the HFD + 5%C and HFD + Veh groups. Moreover, acidic activated charcoal significantly improved HFD-induced hyperinsulinemia, insulin resistance, WAT hypertrophy and intestinal length shortening and could reduce cecum volume to levels comparable to the Con group.

In order to validate the findings of the first study and clarify which concentrations of activated charcoal exhibited the highest anti-obesity properties, we investigated progressively higher doses of the material. Our results showed that 4.5% activated charcoal was the most effective in improving the phenotypic changes induced by long-term HFD feeding without any detrimental effects. This information might be applicable to humans in future food additives to prevent obesity.

In the initial study and dose-dependent study, liver TG levels did not increase in the HFD + Veh group. In this study, inulin, dextrin, and raffinose were used as a vehicle to prevent constipation by charcoal. It was documented that inulin could attenuate hepatic TG accumulation in several mouse models, such as high-fat/high-sucrose treated mice and high-cholesterol treated mice (14, 15). Additionally, raffinose could ameliorate hepatic lipid accumulation in high cholic acid treated rats (16). The absence of marked TG accumulation may be associated with the action of inulin and raffinose to attenuate hepatic steatosis.

According to the metabolomic analysis, large amounts of BAs were excreted into the feces by inhibiting their reabsorption in the intestine, and so altered amounts of BAs in the enterohepatic circulation were detected in the liver. As a compensatory response, *Cyp7a1*, a rate-limiting enzyme for *de novo* BA synthesis from cholesterol, may have been increased by the charcoal; a sufficient supply of cholesterol is necessary to synthesize BAs. The intestinal absorption of dietary cholesterol requires emulsification by BAs (17). A large amount of BAs were combined with activated charcoal and excreted through the feces, which demonstrated altered digestion and absorption of cholesterol in the intestine. These changes may increase *Hmgcs1* and *Hmgcr*.

Prior studies have found that FXR disruption and activated TGR5 in the intestine, which are closely associated with intestinal BA signaling and nutrient homeostasis, produce an anti-obesity effect and improve glucose metabolism (18–22). The inconsistency of changes in intestinal FXR/TGR5 between the two independent experiments prompted us to conclude that FXR/TGR5 was not a primary reason explaining the anti-obesity effect of acidic activated charcoal. The charcoal also absorbed BAs, cholesterol, and neutral lipids in the small intestine, thereby enhancing the excretion of fat into the feces and preventing BW gain.

It was earlier reported that obese patients were in a state of persistent chronic inflammation, which mediated the development of obesity-related diseases, especially type 2 diabetes (23, 24). In particular, adipose expansion induces the adipose inflammation related to insulin resistance (25–27). Indeed, the expression levels of *Il1b* and *Ccl2* in the HFD + 5%C group were significantly decreased vs. the HFD + Veh group. Although no remarkable differences were observed for *Cd68* or *Tnf*, the HFD + 5%C group showed a decreasing trend. In the dose-dependency experiment, the mRNA levels of *Cd68*, *Ccl2*, *Tgfb1*, and *Col1a1* all decreased with increasing levels of acidic activated charcoal. This indicated that the charcoal alleviated the adipose microinflammation caused by obesity, with larger doses of the material being more effective.

Since the activated charcoal was a powder and was not digested by the animals, we were concerned about pneumoconiosis and damage to the digestive tract (28). However, there were no visible differences of the lungs and gastrointestinal tract, and inflammation-related gene levels were not increased in the HFD + 5%C compared with HFD + Veh groups. Based on those findings, we consider the oral administration of acidic activated charcoal to be relatively safe, although further long-term studies are required to confirm its safety and applicability in humans. Moreover, because activated charcoal can absorb lipids, it may disrupt the absorption of fats, fat-soluble vitamins, and folic acid (29–31). As the next step, extended experiments will be mandatory to monitor for deficiencies in essential FAs and fat-soluble vitamins. Acidic activated charcoal can absorb not only lipids, but also toxic contaminants in foods, and metabolites from microbiota, such as indole acetate and lithocholic acid. These metabolites are ligands for the aryl hydrocarbon receptor and pregnane X receptor, which modulate intestinal mucosal immunity (32). It is of great interest whether acidic activated charcoal can modulate the crosstalk between metabolites, the microbiome, epithelial cells, and immune cells.

## Conclusion

Acidic activated charcoal improved HFD-induced obesity and insulin resistance without any serious adverse effects. These beneficial effects were likely due to modulating lipid absorption and altered FA/BA metabolism.

## Data Availability Statement

The raw data supporting the conclusions of this article will be made available by the authors, without undue reservation.

## Ethics Statement

The animal study was reviewed and approved by the Shinshu University School of Medicine.

## Author Contributions

NT contributed to conception and design of the study. HY, YI, and TY made activated charcoal and provided it. XZ, PD, XW, ZZ, XH, TN, TK, and NT conducted animal experiments, collected the data, and performed statistical analysis. KT, CH, and MN performed metabolomic analysis. JN supervised histological analysis. XZ, TK, and NT wrote the manuscript. All authors contributed to manuscript revision, read, and approved the submitted version.

## Conflict of Interest

HY and TY were employed by Sumi Plus Lab Co., Ltd. YI was employed by Ina Carbonization Laboratory Co., Ltd. KT and CH were employed by LSI Medience Corporation. The remaining authors declare that the research was conducted in the absence of any commercial or financial relationships that could be construed as a potential conflict of interest.

## Publisher’s Note

All claims expressed in this article are solely those of the authors and do not necessarily represent those of their affiliated organizations, or those of the publisher, the editors and the reviewers. Any product that may be evaluated in this article, or claim that may be made by its manufacturer, is not guaranteed or endorsed by the publisher.

## Abbreviations

Acta2: actin alpha 2
ALT: alanine aminotransferase
Asbt: apical sodium-bile acid transporter
AST: aspartate aminotransferase
AUC: area under the receiver operating characteristic curve
BA: bile acid
BAT: brown adipose tissue
BW: body weight
Ccl2: chemokine (C-C motif) ligand 2
Cd68: cluster of differentiation 68
Col1a1: collagen, type I, alpha 1
Cyp7a1: cholesterol 7 alpha-hydroxylase
DCA: deoxycholic acid
eWAT: epididymal white adipose tissue
FA: fatty acid
Fabp6: fatty acid-binding protein 6
Fgf15: fibroblast growth factor 15
FXR: farnesoid X receptor
GTT: glucose tolerance test
HFD: high-fat diet
Hmgcr: hydroxymethylglutaryl-CoA reductase
Hmgcs1: hydroxymethylglutaryl-CoA synthase 1
HOMA-IR: homeostatic model assessment for insulin resistance
Il1b: interleukin 1 beta
ITT: insulin tolerance test
NEFA: non-esterified fatty acid
PL: phospholipid
qPCR: quantitative polymerase chain reaction
SEM: standard error of the mean
Shp: small heterodimer partner
TBA: total bile acid
T-Chol: total cholesterol
TG: triglyceride
Tgfb1: transforming growth factor beta 1
TGR5: Takeda G protein-coupled receptor 5
Tnf: tumor necrosis factor alpha
TSH: thyroid-stimulating hormone.
