# Supplementary material for: Acidic Activated Charcoal Prevents Obesity and Insulin Resistance in High-Fat Diet-Fed Mice
Source: Front Nutr. 2022 May 12;9:852767. doi: 10.3389/fnut.2022.852767 (PMC9134190; doi:10.3389/fnut.2022.852767)
Supplement: Supplementary file 1 [file Data_Sheet_1.PDF]

Supplementary information

Methods

Metabolomic analysis

Approximately 80 mg of tissue were transferred to a tissue disruptor tube supplied by Yasui Kikai (Osaka, Japan) and shaken with iron cones cooled in liquid nitrogen. The tissue powder was suspended with 1 mL of water, and then mixed with 2 mL of methanol and 2 mL of chloroform. After mixing with a shaker for 15 min, the sample was centrifuged at 1000 × *g* for 10 min. The supernatant was transferred to a 15 mL Falcon tube and dried under a nitrogen stream at 40°C. The dried residue of the supernatant was dissolved with 200 µL of a 10% acetonitrile/90% water solution, and then analyzed using capillary electrophoresis-mass spectrometry (CE/MS) and liquid chromatography-mass spectrometry (LC/MS). Twelve samples were analyzed twice for every six platforms, i.e., CE/MS and LC/MS evaluated by both positive and negative polarities. The average of two data sets was calculated to determine expression values. Quality control (QC) was prepared by pooling 12 samples and analyzed every six measurements. Consequently, five QCs were analyzed in every run. The coefficient of variance (CV%) of every metabolite was calculated using five QCs, and metabolites with a CV more than 50 % were omitted from the data table.

CE/MS experiments were performed using an Agilent CE capillary electrophoresis system (Agilent Technologies, Waldbronn, Germany) and an Agilent 6520 QTOF system (Agilent Technologies, Palo Alto, CA). Cationic separation was conducted in fused-silica capillaries (50 µm i.d. 100 cm total length) filled with 1 mol/L formic acid, and anionic separation was conducted with 20 mmol/L ammonium acetate and 20 mmol/L ammonium formate (pH 10) as the electrolyte. Sample solutions were injected at 50 mbar for 10 s, and a voltage of 30 kV was applied. The capillary temperature was not controlled (i.e., maintained at room temperature), and the temperature of the sample tray was maintained at 4°C using an external thermostatic cooler. The sheath liquid (methanol/water, 50% v/v) was delivered at 8 µL/min. Electrospray ionization time-of-flight mass spectrometry (ESI-TOF-MS) was conducted in both positive (cationic) and negative (anionic) ion modes. The capillary voltage was set to 3 kV, and the flow rate of nitrogen gas (heater temperature: 300 °C) was set to 10 psi. Exact mass data were acquired over a 60-1200 m/z range in full scan mode.

LC/MS analyses were performed using an Agilent 1290 series UPLC system equipped with a 6520 quadrupole TOF system (Agilent Technologies, Palo Alto, CA) operated by Masshunter Workstation B.04.01 software. The analytical column was a CAPCELL PAK C18 IF 2.0 mm I.D. × 50 mm, 2 µm (Osaka Soda, Osaka, Japan). The mobile phase consisted of two solvents: eluent A was water/ammonium acetate (5 mmol/L) and eluent B was acetonitrile. Metabolites were eluted at a flow rate of 0.2 mL/min at 40°C with a linear gradient of 10-100% of eluent B over 10 min with a further 5 min hold at 100% of eluent B. Injection volume was 10 µL. Mass spectrometric analysis was performed in both positive and negative ionization modes with a scan rate of 2 spectra/s, mass range of 60-1200 m/z, capillary voltage of 3500

V, and fragmentor setting of 120 V. The pressure of the nebulizer was 40 psi, gas temperature was 350°C, and continuous gas flow was 8 L/min.

Raw data obtained from the mass spectrometer were converted to CSV format using a Mass Hunter Export (Agilent Technologies). The converted CSV data comprised m/z, retention time, and intensity. Data processing was performed in the following sequence: 1) peak picking (identifying peak positions and areas), 2) bias correction of retention time and m/z, 3) peak alignment, 4) noise reduction, 5) bias correction of peak intensity, and 6) peak identification. All processes were performed using Marker analysis. All peak areas were divided by the area of the internal standard to avoid injection-volume bias and mass spectrometry detector sensitivity bias among multiple measurements, and were normalized by wet tissue weight. The peak areas with a signal-to-noise ratio of less than three were converted to zero. Thereafter, isotopic, fragment, and adduct ions were eliminated, and the peak datasets were compared across the samples and aligned according to m/z and retention time. Noise peaks in the samples were detected by comparisons with blank preparation samples. The peaks found in the samples were identified based on the matched m/z values and normalized retention times of the 1379 standards that were purchased from reagent manufacturers; thus, all standards were previously analyzed with the same platforms. When several metabolites were simultaneously identified, we conducted tandem mass spectrometry (MS/MS) analysis to confirm the structures by matching the fragmentation patterns with those of the candidates. Although all metabolites were quantified separately, the sum of the quantified values of several isomers, including citrate and isocitrate, were counted as a single marker due to the low separation of the peaks. The recovery and stability of each metabolite were not assessed.

Supplementary Figure 1. Gross appearance of mice treated with acidic activated charcoal-containing high-fat diet and serum thyroid-stimulating hormone (TSH) levels.

(A) The limbs, fur and tail of the acidic activated charcoal diet-treated mice.
(B) Serum TSH levels.

Data are expressed as the mean ± SEM. Statistical analysis was performed using Two-tailed Student's t-tests or a one-way analysis of variance (ANOVA) with the Bonferroni's correction. $^{**}P < 0.01$ between the HFD+Veh group and the HFD+C group. $^{\#\#}P < 0.01$ between the HFD+Veh group and the Con group.

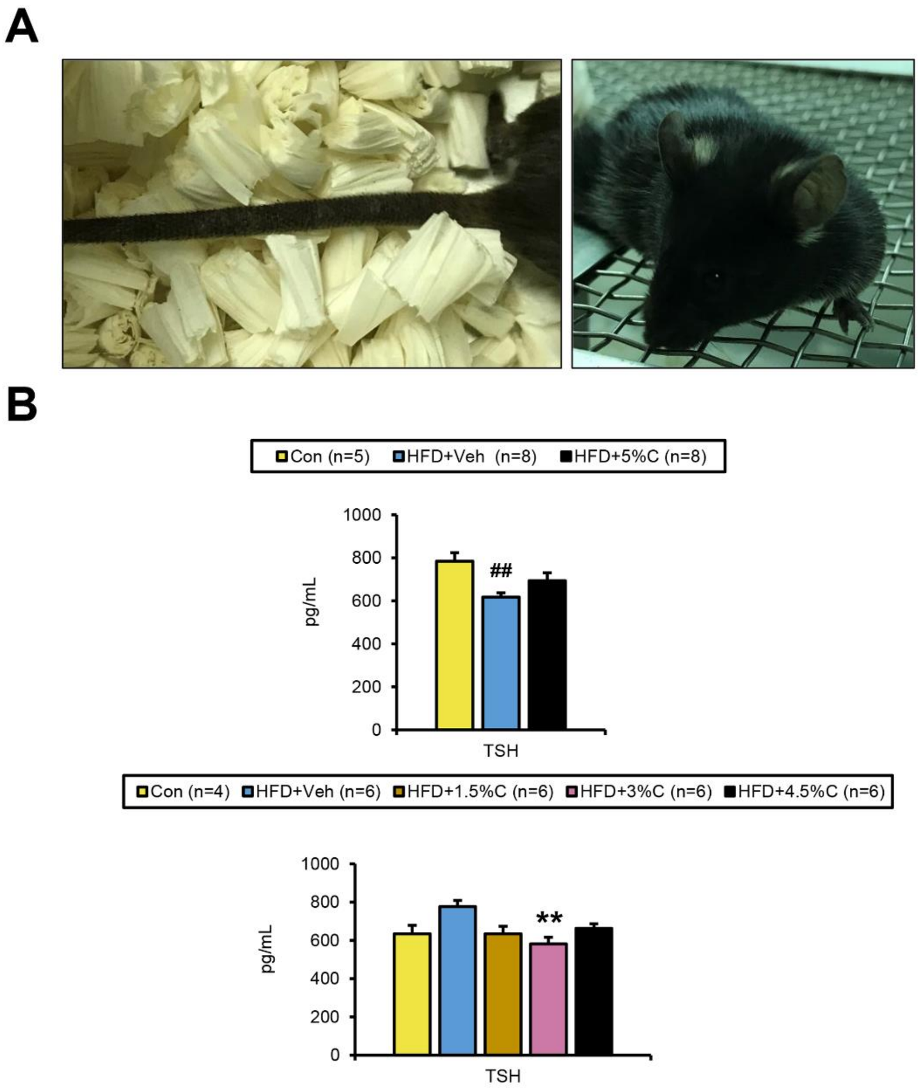

Supplementary Figure 2. The pancreatic functions appear to be maintained in the acidic activated charcoal-treated mice.

(A) Histological appearance of pancreas. The sections were stained with hematoxylin and eosin. Scale bars = 100 μm (x200 magnification).
(B) The relative islet area, expressed as a percentage of the total area of pancreatic parenchyma, and the pancreatic gene expression levels of *Ins2* (insulin 2).

Data are expressed as the mean ± SEM. Statistical analysis was performed using two-tailed Student's t-tests.

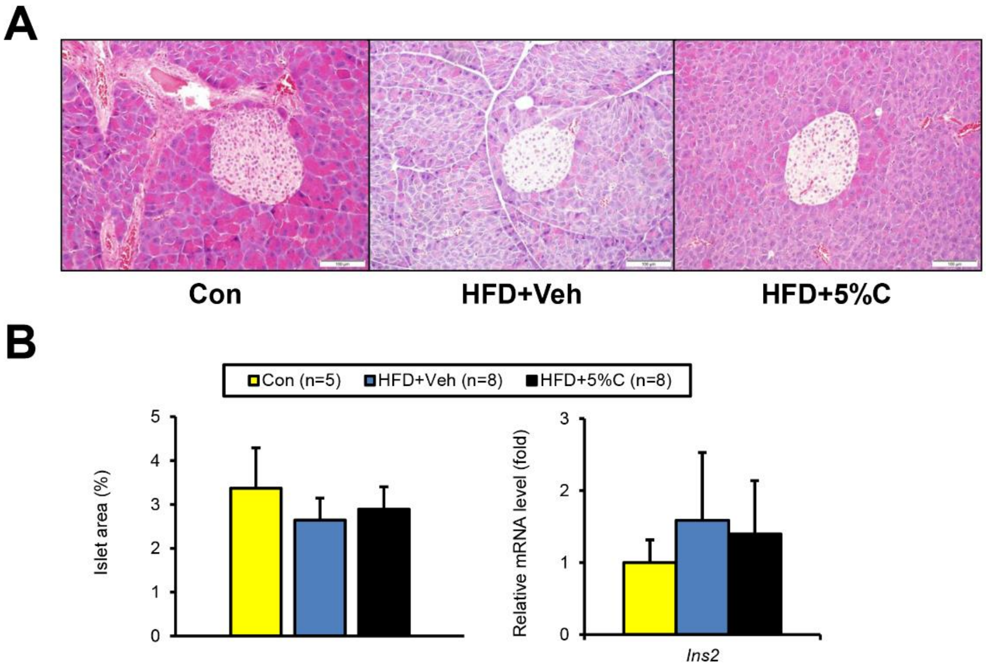

Supplementary Figure 3. Changes in liver gene expression level and liver lipid content by activated charcoal treatment.

(A) Histological appearance of liver. The sections were stained with hematoxylin and eosin. Scale bars = 150 μm (x200 magnification).
(B-D) The mRNA levels of genes related to FA/TG metabolism.
(E) The mRNA levels of genes related to inflammation.
(F) The mRNA levels of genes related to fibrosis.
(G) Quantification of hepatic lipids.

Data are expressed as the mean ± SEM. Statistical analysis was performed using two-tailed Student's t-tests. *$P < 0.05$ between the HFD+Veh group and the HFD+5%C group. $^{\#}P < 0.05$ and $^{\#\#}P < 0.01$ between the HFD+Veh group and the Con group.

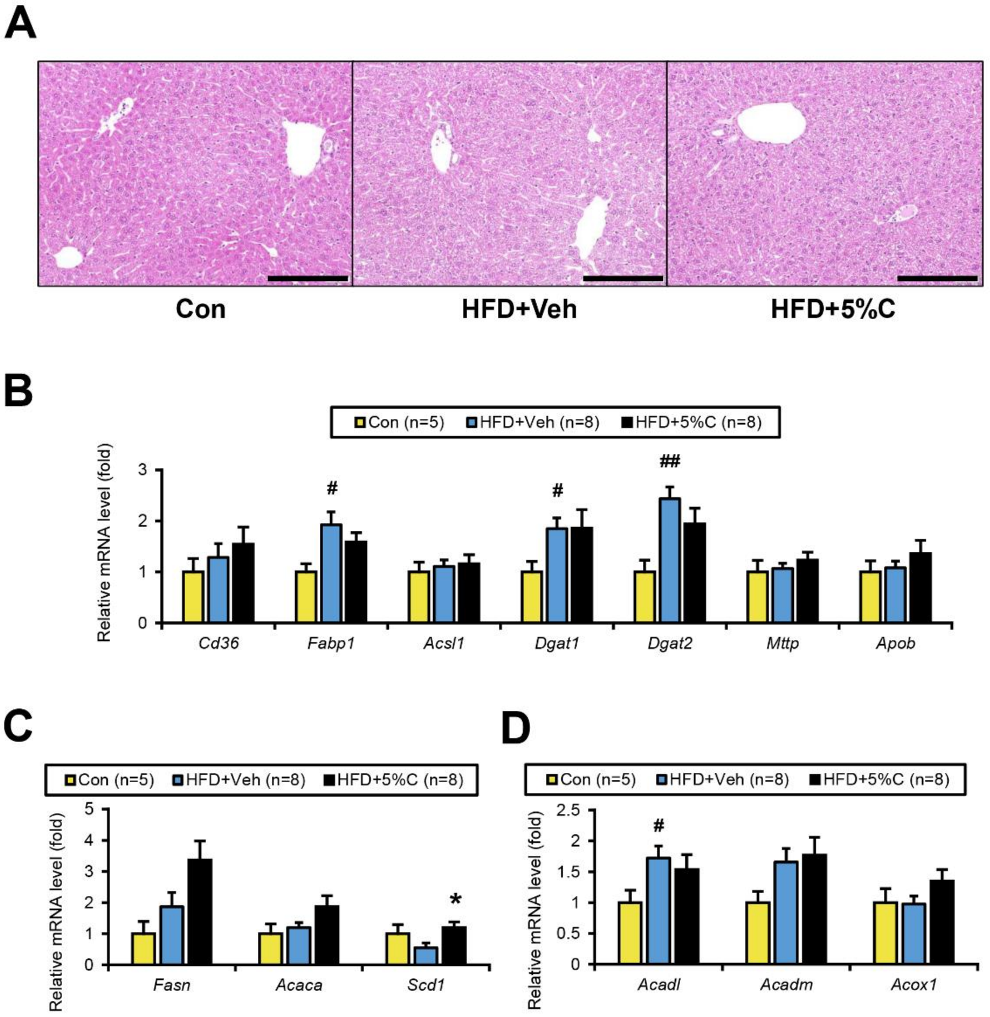

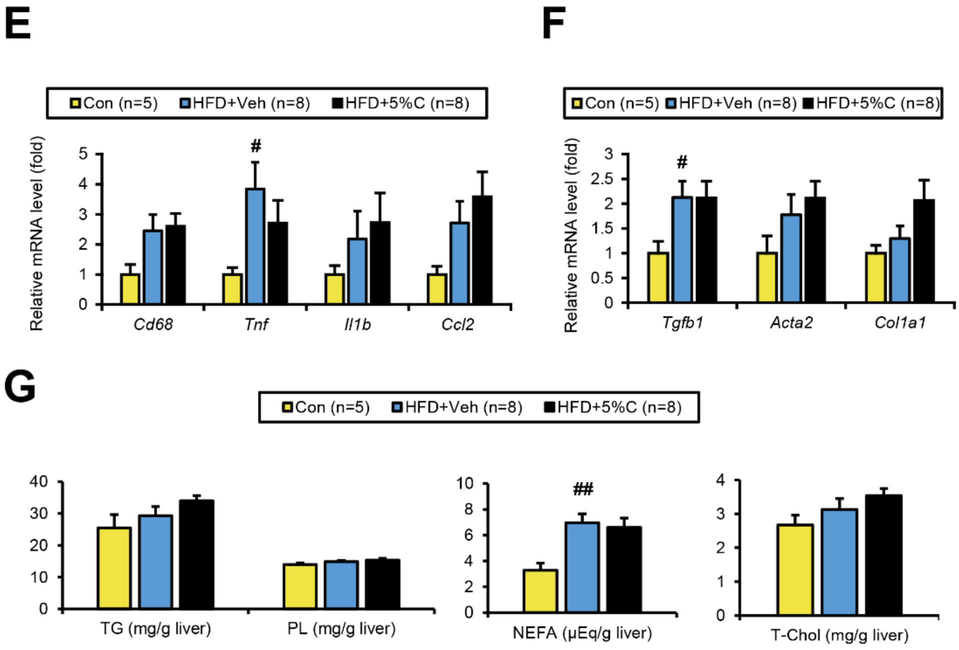
E
Con (n=5)
HFD+Veh (n=8)
HFD+5%C (n=8)
Relative mRNA level (fold)
0
1
2
3
4
5
#
Cd68
Tnf
Il1b
Ccl2
F
Con (n=5)
HFD+Veh (n=8)
HFD+5%C (n=8)
Relative mRNA level (fold)
0
0.5
1
1.5
2
2.5
3
#
Tgfb1
Acta2
Col1a1
G
Con (n=5)
HFD+Veh (n=8)
HFD+5%C (n=8)
0
10
20
30
40
TG (mg/g liver)
PL (mg/g liver)
0
2
4
6
8
10
##
NEFA (μEq/g liver)
0
1
2
3
4
T-Chol (mg/g liver)

Supplementary Figure 4. Changes in eWAT gene expression level by activated charcoal treatment.

(A) The mRNA levels of genes related to FA uptake.
(B) The mRNA levels of genes related to FA synthesis.
(C) The mRNA levels of genes related to fat burning.
(D) The mRNA levels of genes related to browning.
(E) The mRNA levels of genes related to lipolysis.

Data are expressed as the mean ± SEM. Statistical analysis was performed using two-tailed Student's t-tests. *$P < 0.05$ between the HFD+Veh group and the HFD+5%C group. #$P < 0.05$ between the HFD+Veh group and the Con group.

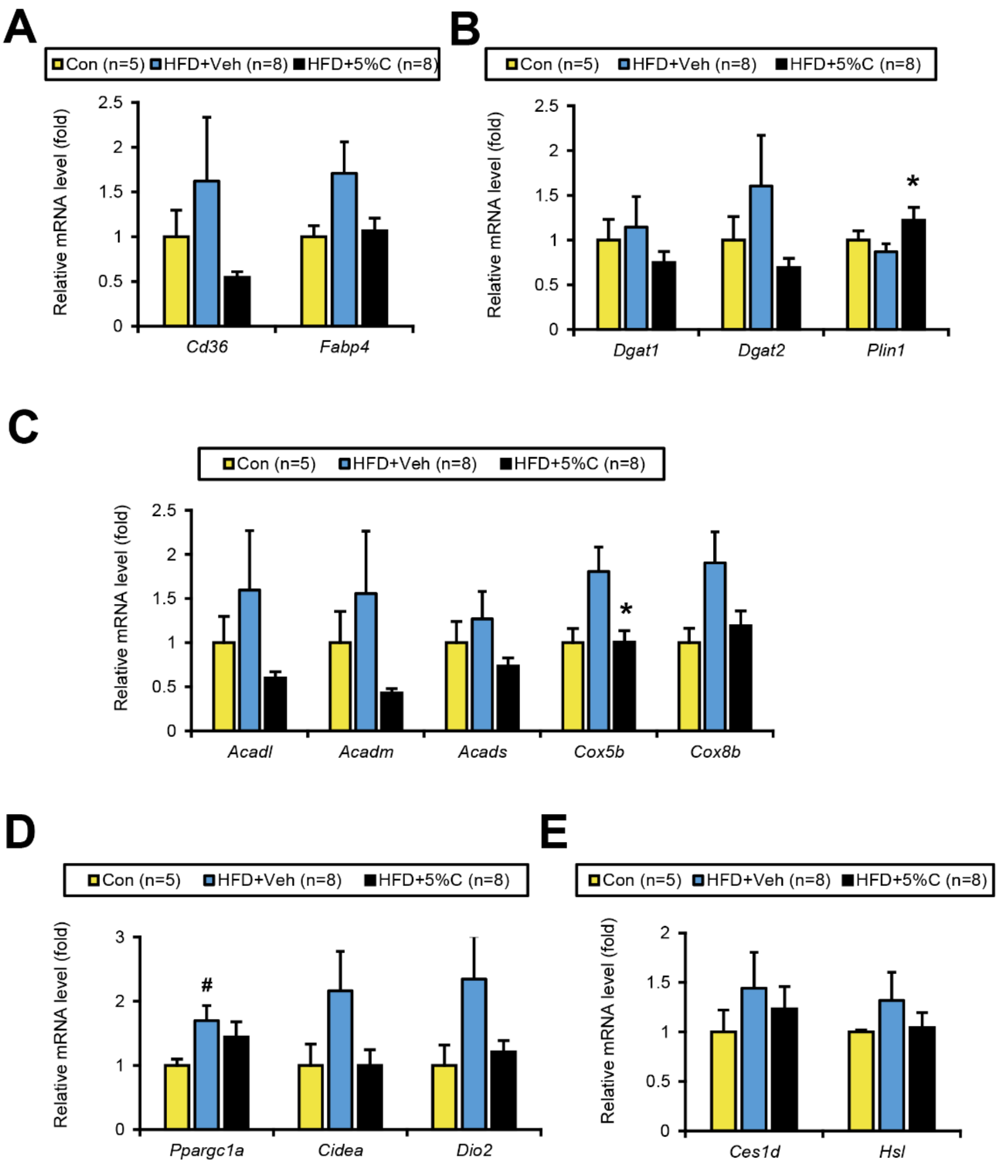

Supplementary Figure 5. Changes in BAT and the gene expression level by activated charcoal treatment.

(A) Histological appearance of BAT. The sections were stained with hematoxylin and eosin. Scale bars = 150 μm (x200 magnification).
(B) Gene expression levels in BAT.

Data are expressed as the mean ± SEM. Statistical analysis was performed using two-tailed Student's t-tests. $^{\#\#}P < 0.01$ between the HFD+Veh group and the Con group.

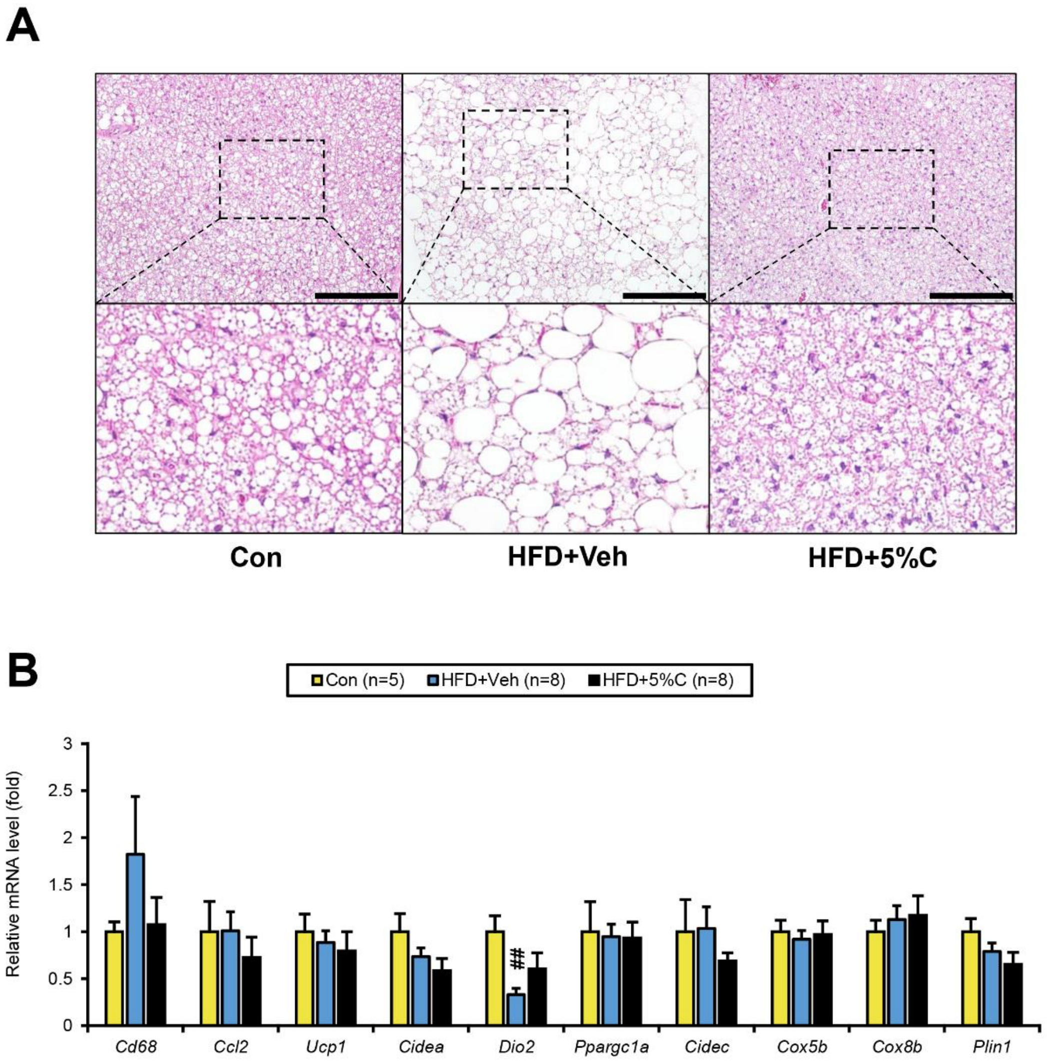

Supplementary Figure 6. Changes in inflammatory signaling of the lung and digestive tract by activated charcoal treatment.

(A) The mouse bedding in the cage turned black by the charcoal treatment.
(B) Gene expression levels in the lung.
(C) Gene expression levels in the stomach.
(D) Gene expression levels in the upper small intestine.
(E) Gene expression levels in the lower small intestine.
(F) Gene expression levels in the large intestine.

Data are expressed as the mean ± SEM. Statistical analysis was performed using two-tailed Student's t-tests. $^{\#}P < 0.05$ between the HFD+Veh group and the Con group.

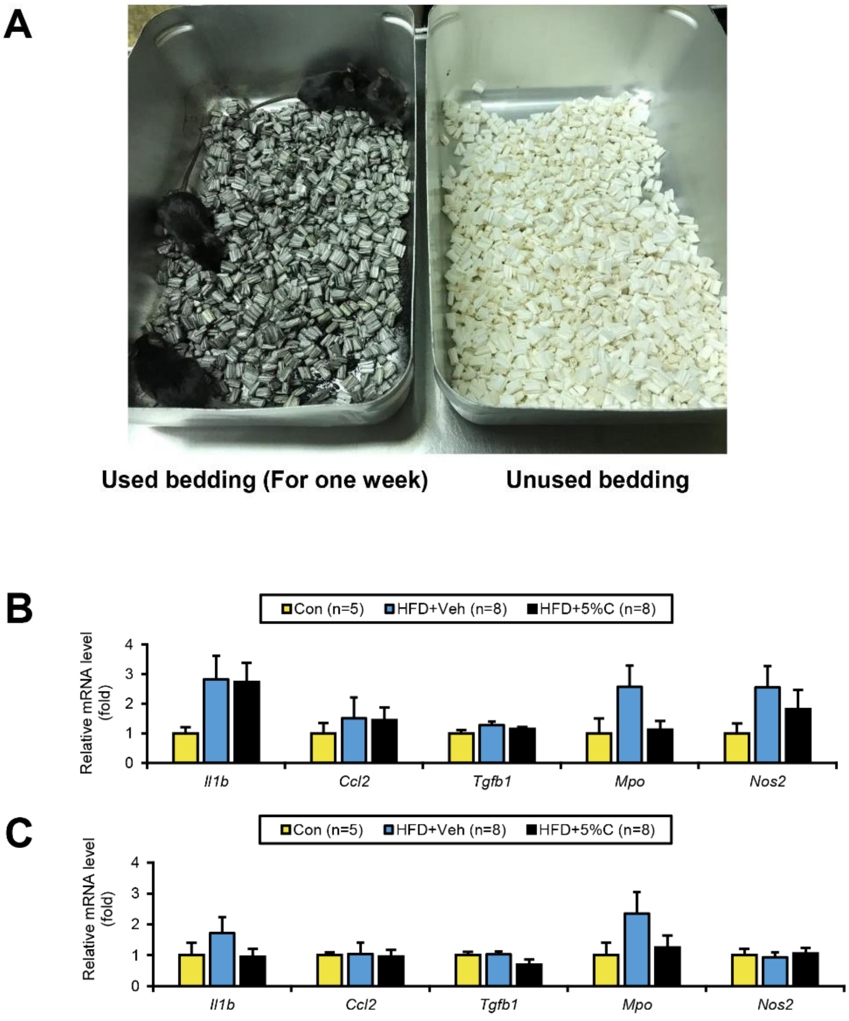

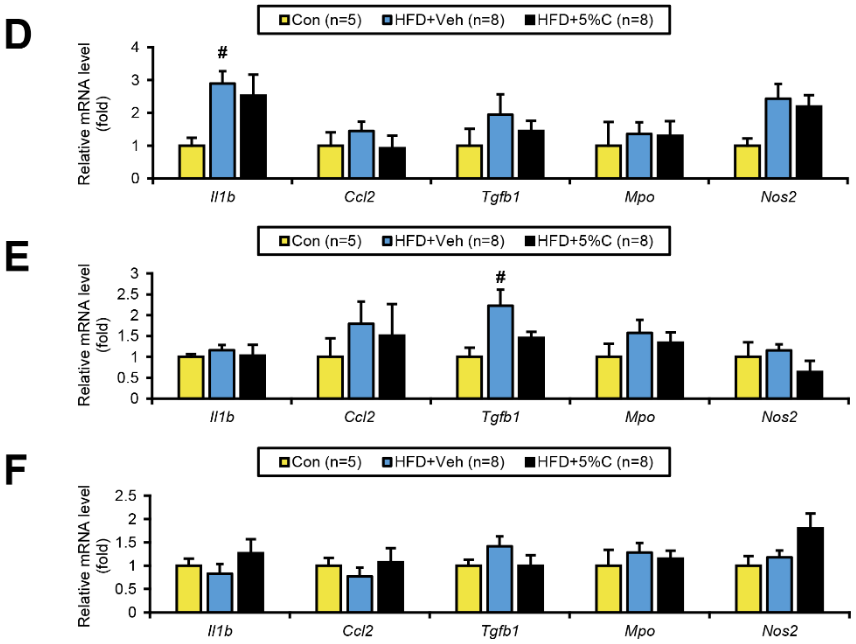
D
Con (n=5)
HFD+Veh (n=8)
HFD+5%C (n=8)
Relative mRNA level (fold)
0
1
2
3
4
#
Il1b
Ccl2
Tgfb1
Mpo
Nos2
E
Con (n=5)
HFD+Veh (n=8)
HFD+5%C (n=8)
Relative mRNA level (fold)
0
0.5
1
1.5
2
2.5
3
#
Il1b
Ccl2
Tgfb1
Mpo
Nos2
F
Con (n=5)
HFD+Veh (n=8)
HFD+5%C (n=8)
Relative mRNA level (fold)
0
0.5
1
1.5
2
2.5
Il1b
Ccl2
Tgfb1
Mpo
Nos2

Supplementary Figure 7. Quantification of hepatic lipids in the second dose-dependent experiment.

Data are expressed as the mean ± SEM. Statistical analysis was performed using a one-way analysis of variance (ANOVA) with the Bonferroni's correction. $^{\#\#}P < 0.01$ between the HFD+Veh group and the Con group.

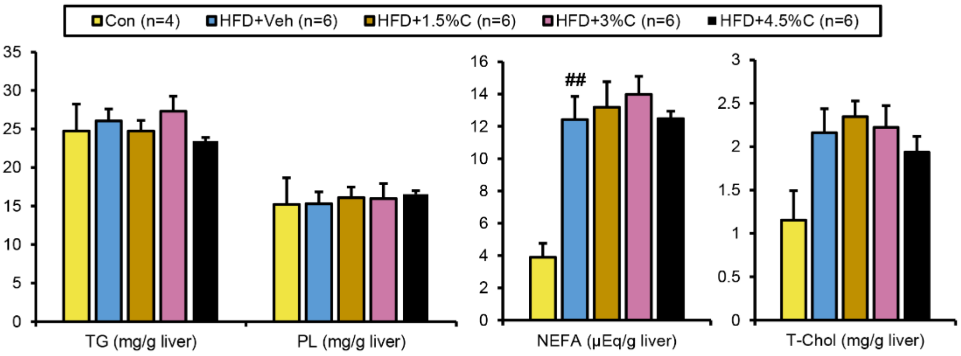

Supplementary Table 1. The ingredients of the control diet (in 100g)

General ingredients		Vitamins		Minerals		Amino acid	
Water (g)	7.9	Vitamin A (IU)	1283	Calcium (g)	1.07	Isoleucine (g)	0.89
Crude protein (g)	23.1	Vitamin D3 (IU)	137	Phosphorus (g)	0.83	Leucine (g)	1.74
Crude fat (g)	5.1	Vitamin E (mg)	9.1	Magnesium (g)	0.24	Lysine (g)	1.24
Crude ash (g)	5.8	Vitamin K3 (mg)	0.04	Sodium (g)	0.19	Methionine (g)	0.44
Crude fiber (g)	2.8	Vitamin B1 (mg)	2.05	Potassium (g)	0.9	Cystine (g)	0.36
Soluble nitrogen-free substance (g)	55.3	Vitamin B2 (mg)	1.1	Iron (mg)	10.6	Phenylalanine (g)	1.04
Calories (kcal)	359	Vitamin C (mg)	4	Aluminum (mg)	2.1	Tyrosine (g)	0.68
		Vitamin B6 (mg)	0.87	Copper (mg)	0.78	Threonine (g)	0.89
		Vitamin B12 (μg)	5.5	Zinc (mg)	4.89	Tryptophan (g)	0.28
		Inositol (mg)	439	Cobalt (mg)	0.1	Valine (g)	1.08
		Biotin (μg)	27	Manganese (mg)	4.84	Arginine (g)	1.42
		Pantothenic acid (mg)	2.45	Ca / P	1.29	Histidine (g)	0.6
		Niacin (mg)	10.61	Ca / Mg	4.5	Alanine (g)	1.2
		Choline (g)	0.18	K / Na	4.77	Aspartic acid (g)	2.14
		Folic acid (mg)	0.17			Glutamic acid (g)	3.99
						Glycine (g)	1.18
						Proline (g)	1.31
						Serin (g)	1.11

204

Supplementary Table 2. The ingredients of high-fat diet (in 100g)

General ingredients		Vitamins		Minerals	
Protein (Casein, Lactic, 30 Mesh) (g)	25.84	Choline Bitartrate (mg)	25.84	Sucrose, Fine Granulated (g)	1.16
Protein (Cystine, L) (g)	0.39	Sucrose, Fine Granulated (mg)	10.13	Potassium Citrate, Monohydrate (g)	2.13
Carbohydrate (Lodex 10) (g)	16.15	Vitamin E Acetate, 50% (mg)	12.92	Calcium Phosphate, Dibasic (g)	1.68
Carbohydrate (g)	9.41	Niacin (a.k.a. B3) (mg)	3.88	Calcium Carbonate, Light, USP (g)	0.71
Fiber (Solka Floc, FCC200) (g)	6.46	Biotin, 1% (mg)	2.58	Sodium Chloride (mg)	334.69
Fat (Lard) (g)	31.66	Pantothenic Acid, d, Calcium (a.k.a. B5) (mg)	2.07	Magnesium Sulfate, Heptahydrate (mg)	332.88
Fat (Soybean Oil, USP) (g)	3.23	Vitamin D3, 100,000 IU/gm (mg)	1.29	Magnesium Oxide, Heavy, DC USP (mg)	54.14
Dye (mg)	6.46	Vitamin B12, 0.1% Mannitol (mg)	1.29	Ferric Citrate (mg)	27.14
		Vitamin A Acetate, 500,000 IU/gm (mg)	1.03	Manganese Carbonate Hydrate (mg)	15.83
		Pyridoxine HCl (a.k.a. B6) (mg)	0.90	Zinc Carbonate (mg)	7.24
		Riboflavin (a.k.a. B2) (mg)	0.78	Chromium Potassium Sulfate (mg)	2.52
		Thiamine HCl (a.k.a. B1) (mg)	0.78	Copper Carbonate (mg)	1.36
		Folic Acid (mg)	0.26	Ammonium Molybdate Tetrahydrate (mg)	0.39
		Menadione Sodium Bisulfite (mg)	0.10	Sodium Fluoride (mg)	0.26
				Sodium Selenite (mg)	0.06
				Potassium Iodate (mg)	0.06

Supplementary Table 3. Primer pairs used for qPCR analysis

Gene	Accession #	Primer sequence (5'-3')
18S rRNA	NR_003278	F 5'-CACGGACAGGATTGACAGATTG-3'
		R 5'-CAGACAAATCGCTCCACCAA-3'
Acaca	NM_133360	F 5'-GGGCACAGACCGTGGTAGTT-3'
		R 5'-CAGGATCAGCTGGGATACTGAGT-3'
Acadl	NM_007381	F 5'- TTTCCGGGAGAGTGTAAGGA-3'
		R 5'- ACTTCTCCAGCTTTCTCCCA-3'
Acadm	NM_007382	F 5'-TGCTTTTGATAGAACCAGACCTACAGT-3'
		R 5'-CTTGGTGCTCCACTAGCAGCTT-3'
Acads	NM_007383	F 5'-CTCCACAGCTAACCTCATCTTTG-3'
		R 5'-GGGTTTGCATGGCTATTTTG-3'
Acox1	NM_015729	F 5'-TGGTATGGTGTCGTACTTGAATGAC-3'
		R 5'-AATTTCTACCAATCTGGCTGCAC-3'
Acsl1	NM_007981	F 5'-TCCTACGGCAGTGATCTGGTG-3'
		R 5'-GGTTGCCTGTAGTTCCACTTGTG-3'
Acta2	NM_007392	F 5'-ACTGGGACGACATGGAAAAG-3'
		R 5'-GTTCAGTGGTGCCTCTGTCA-3'
Apob	NM_009693	F 5'-TCACCCCCGGGATCAAG-3'
		R 5'-TCCAAGGACACAGAGGGCTTT-3'
Asbt	NM_011388	F 5'-ACTGTACCAAAGTGCCTGGA-3’
		R 5'-GCTACTGTTCGGCACCTGT-3’
Bsep	NM_021022	F 5'-TCAGTTCCTCCGTTCAAACATTG-3'
		R 5'-TCTCTTTGGTGTTGTCCCCATA-3'
Ccl2	NM_011333	F 5'-AGGTCCCTGTCATGCTTCTG-3'
		R 5'-GGGATCATCTTGCTGGTGAA-3'
Cd36	NM_007643	F 5'-CCAAATGAAGATGAGCATAGGACAT-3'
		R 5’-GTTGACCTGCAGTCGTTTTGC-3’
Cd68	NM_001291058	F 5'-GAGGTTGTGACGGTACCCAT-3’
		R 5’-ACATTGTATTCCACCGCCAT-3’
Ces3	NM_053200	F 5'-TGGTATTTGGTGTCCCATCA-3'
		R 5'-GCTTGGGCGATACTCAAACT-3'
Cidea	NM_007702	F 5'-CAGTGATTTAAGAGACGCGG-3'
		R 5'-TCTGCAATCCCATGAATGTC-3'
Cidec	NM_178373	F 5'-GCCACGCGGTATTGCCAGGA-3'
		R 5'-GGGTCTCCCGGCTGGGCTTA-3'
Col1a1	NM_007742	F 5’-ACATGTTCAGCTTTGTGGACC-3’
		R 5’-TAGGCCATTGTGTATGCAGC-3’
Cox5b	NM_009942	F 5'-CGTCCATCAGCAACAAGAGA-3'
		R 5'-ATCGCTGACTCTCGCCTTT-3'
Cox8b	NM_007751	F 5'-TGCGAAGTTCACAGTGGTTC-3'
		R 5'-AGCCAACGACTATGGCTGAG-3'
Cyp7a1	NM_007824	F 5'-CGCATGTTTCTCAACGACACA-3'
		R 5'-ATGCCCAGAGGATCACAAGGT-3'
Dgat1	NM_010046	F 5'-CTGCTACGACGAGTTCTTGAGA-3'
		R 5'-GATAGTAGGGACCATCCACTGTTG-3'
Dgat2	NM_026384	F 5'-GCTTCGCGAGTACCTGATGT-3'

		R 5'-CACCACGATGATGATAGCATTG-3'
Dio2	NM_010050	F 5'-TCCTAGATGCCTACAAACAGGTTA-3'
		R 5'-CGGTCTTCTCCGAGGCATAA-3'
Fabp1	NM_017399	F 5'-GCAGAGCCAGGAGAACTTTGAG-3'
		R 5'-TTTGATTTTCTTCCCTTCATGCA-3'
Fabp4	NM_024406	F 5'-TTTCCTTCAAACTGGGCGTG-3'
		R 5'-AGGGTTATGATGCTCTTCACCTTC-3'
Fabp6	NM_008375	F 5'-GTTCATGAAGCGCCTGGGTCT-3'
		R 5'-CTGGGACCAGGTGAAGTCCT-3'
Fasn	NM_007988	F 5’-ATCCTGGAACGAGAACACGATCT-3’
		R 5’-AGAGACGTGTCACTCCTGGACTT-3’
Fgf15	NM_008003	F 5'-ATGGCGAGAAAGTGGAACGG-3’
		R 5'-CTGACACAGACTGGGATTGCT-3’
Fxr	NM_009108	F 5'-GATTTGGAATCGTACTCCCCATAC-3'
		R 5'-GAAGCCCAGGTTGGAATAGTAAGA-3'
Hmgcr	NM_008255	F 5'-TGTGGTTTGTGAAGCCGTCAT-3'
		R 5'-CGTCAACCATAGCTTCCGTAGTT-3'
Hmgcs1	BC029693	F 5'-CAGGAAATGCCAGACCTACAGG-3'
		R 5'-AGTCATAGGCATGCTGCATGTG-3'
Hsl	NM_010719	F 5'-GAGCGCTGGAGGAGTGTTTT-3'
		R 5'-TGATGCAGAGATTCCCACCTG-3'
Il1b	NM_008361	F 5'-TGAAGCAGCTATGGCAACTG-3'
		R 5'-AGGTCAAAGGTTTGGAAGCA-3'
Mpo	NM_010824	F 5'-CTCCTCACCAACCGCTCC -3'
		R 5'-TGCTCTCGAACAAAGAGGGT-3'
Mttp	NM_008642	F 5'-GAGCGGTCTGGATTTACAACG-3'
		R 5'-GTAGGTAGTGACAGATGTGGCTTTTG-3'
Nos2	NM_010927	F 5'-GAGCTCGGGTTGAAGTGGTATG-3'
		R 5'-TGTGTGTCACAGAAGTCTCGAACTC-3'
Ppargc1a	NM_008904	F 5'-TCTCAGTAAGGGGCTGGTTG-3'
		R 5'-AGCAGCACACTCTATGTCACTC-3'
Plin1	NM_175640	F 5'-TGAAGCAGGGCCACTCTC-3'
		R 5'-GACACCACCTGCATGGCT-3'
Scd1	NM_009127	F 5'-AGATCTCCAGTTCTTACACGACCAC-3'
		R 5'-CTTTCATTTCAGGACGGATGTCT-3'
Shp	NM_011850	F 5'-TGGCCTCTACCCTCAAGAACA-3'
		R 5'-CATGTCTTCAAGGAGTTCAGTGATG-3'
Tgfb1	NM_011577	F 5'-GGAGAGCCCTGGATACCAAC-3'
		R 5'-CAACCCAGGTCCTTCCTAAA-3'
Tgr5	NM_174985	F 5'-GAGCGTCGCCCACCACTAGG-3'
		R 5'-CGCTGATCACCCAGCCCCATG-3'
Tnf	NM_013693	F 5'-CCACCACGCTCTTCTGTCTAC-3'
		R 5'-AGGGTCTGGGCCATAGAACT-3'
Ucp1	NM_009463	F 5'-AGGATGGTGAACCCGACAAC-3'
		R 5'-GGCCTTCACCTTGGATCTGA-3'

F, forward sequence; R, reverse sequence.

Acaca, acetyl-coenzyme A carboxylase alpha

Acadl, acyl-coenzyme A dehydrogenase, long-chain

Acadm, acyl-coenzyme A dehydrogenase, medium-chain
Acads, acyl-coenzyme A dehydrogenase, short-chain
Acox1, acyl-coenzyme A oxidase 1, palmitoyl
Acsl1, acyl- coenzyme A synthetase long-chain family member 1
Acta2, actin, alpha 2, smooth muscle, aorta
Apob, apolipoprotein B
Asbt, apical sodium-dependent bile acid transporter
Bsep, bile salt export pump
Ccl2, C-C motif chemokine ligand 2
Cd36, cluster of differentiation 36
Cd68, cluster of differentiation 68
Ces1d, carboxylesterase 1d
Cidea, cell death-inducing DNA fragmentation factor alpha-like effector A
Cidec, cell death-inducing DNA fragmentation factor alpha-like effector C
Col1a1, collagen, type I, alpha 1
Cox5b, cytochrome c oxidase subunit 5B
Cox8b, cytochrome c oxidase subunit 8B
Cyp7a1, cytochrome p450 family 7 subfamily A member 1
Dgat1, diacylglycerol O-acyltransferase 1
Dgat2, diacylglycerol O-acyltransferase 2
Dio2, type 2 iodothyronine deiodinase
Fabp1, fatty acid binding protein 1
Fabp4, fatty acid binding protein 4
Fabp6, fatty acid binding protein 6
Fasn, fatty acid synthase
Fgf15, fibroblast growth factor 15
Fxr, farnesoid X receptor
Hmgcr, hydroxymethylglutaryl-CoA reductase
Hmgcs1, hydroxymethylglutaryl-CoA synthase 1
Hsl, hormone-sensitive lipase
Il1b, interleukin 1 beta
Mpo, myeloperoxidase
Mttp, microsomal triglyceride transfer protein
Nos2, nitric oxide synthase 2
Ppargc1a, peroxisome proliferator-activated receptor gamma coactivator 1-alpha
Plin1, perilipin-1
Scd1, stearoyl-coenzyme A desaturase 1
Shp, small heterodimer partner
Tgfb1, transforming growth factor, beta 1
Tgr5, Takeda G protein-coupled receptor 5
Tnf, tumor necrosis factor alpha
Ucp1, uncoupling protein 1

Supplementary Table 4. Top 20 significantly increased metabolites in cecal content

Top	Rt	Ms	Name	Category	Con Mean (n=4)	HFD+Veh Mean (n=4)	HFD+5%C Mean (n=4)	*P*-value (Veh vs 5%C)	TEST-T (Veh vs 5%C)	Separation (Veh vs 5%C)	Ratio (Veh vs 5%C)
1	8.08	522.355	1-oleoylglycerophosphocholine	Lyso PC	1.0	0.3	2.9	2.6E-05	**	*	9.65
2	7.29	281.247	Ricinoleic acid (18:1-OH)	Fatty acid	1.0	1.0	7.1	6.3E-05	**	*	7.49
3	7.93	496.339	1-palmitoylglycerophosphocholine (16:0)	Lyso PC	1.0	0.5	1.7	1.4E-04	**	*	3.70
4	12.96	187.100	Azelaic acid	Fatty acid metabolism	1.0	0.3	1.0	1.7E-04	**	*	2.82
5	0.64	198.991	D-erythrose 4-phosphate	Pentose phosphate pathway	1.0	3.2	10.8	2.2E-04	**	*	3.32
6	0.62	105.065	2-hydroxybutyric acid	Organic acid	1.0	1.2	2.3	2.3E-04	**	*	1.96
7	5.62	174.053	Indole acetate	Tryptophan metabolism	1.0	0.3	0.7	5.6E-04	**	*	2.39
8	14.69	174.063	Quinaldic acid	Unclassified	1.0	0.2	1.9	5.9E-04	**	*	10.50
9	11.41	213.184	Tridecanoic acid	Fatty acid	1.0	0.9	2.5	6.4E-04	**	*	2.73
10	11.92	327.234	Docosahexaenoic acid (22:6;4,7,10,13,16,19)	Fatty acid	1.0	0.6	3.0	6.9E-04	**	*	4.96
11	8.02	400.342	L-palmitoyl carnitine	Acyl carnitine	1.0	0.6	3.5	8.8E-04	**	*	5.90
12	7.33	123.058	Nicotinamide	Nicotinate and Ncotinamide metabolism	1.0	0.2	0.7	1.4E-03	**	*	3.74
13	9.16	391.288	Deoxycholate	Bile acid	1.0	1.2	2.4	1.5E-03	**	*	1.93
14	4.33	134.058	Indoxyl	Tryptophan metabolism	1.0	1.7	6.6	1.6E-03	**	*	3.86
15	6.92	361.200	11beta-hydroxyandrost-4-ene-3,17-dione	Sterol metabolism	1.0	1.3	3.1	2.0E-03	**	*	2.38
16	7.18	359.190	Adrenosterone	Steroid	1.0	12.9	42.7	2.2E-03	**	*	3.31
17	9.03	391.284	Hyodeoxycholic acid	Bile acid	1.0	0.8	4.1	2.4E-03	**	*	4.92
18	10.59	375.291	Lithocholic acid	Bile acid	1.0	2.8	7.8	2.4E-03	**	*	2.82
19	6.91	514.287	Taurocholate	Bile acid	1.0	0.5	1.6	2.4E-03	**	*	3.37
20	11.88	329.229	Docosapentaenoate (n3 DPA; 22:5n3)	Fatty acid	1.0	0.6	3.8	2.5E-03	**	*	6.27
